# Supplementary material for: Visual impairment and objectively measured physical activity and sedentary behaviour in US adolescents and adults: a cross-sectional study
Source: BMJ Open. 2019 Apr 14;9(4):e027267. doi: 10.1136/bmjopen-2018-027267 (PMC6500295; doi:10.1136/bmjopen-2018-027267)
Supplement: Author's manuscript [file bmjopen-2018-027267.draft_revisions.pdf]

BMJ Open is committed to open peer review. As part of this commitment we make the peer review history of every article we publish publicly available.

When an article is published we post the peer reviewers' comments and the authors' responses online. We also post the versions of the paper that were used during peer review. These are the versions that the peer review comments apply to.

The versions of the paper that follow are the versions that were submitted during the peer review process. They are not the versions of record or the final published versions. They should not be cited or distributed as the published version of this manuscript.

BMJ Open is an open access journal and the full, final, typeset and author-corrected version of record of the manuscript is available on our site with no access controls, subscription charges or pay-per-view fees (http://bmjopen.bmj.com).

If you have any questions on BMJ Open's open peer review process please email info.bmjopen@bmj.com

BMJ Open

Visual impairment and objectively measured physical activity and sedentary behavior in US adolescents and adults

Journal:	*BMJ Open*
Manuscript ID	bmjopen-2018-027267
Article Type:	Research
Date Submitted by the Author:	14-Oct-2018

Complete List of Authors:	Smith, Lee; Anglia Ruskin University - Cambridge Campus, Department of Life Sciences<br>Jackson, Sarah ; University College London, Epidemiology and Public Health;<br>Pardhan, Shahina ; Anglia Ruskin University, Vision and Eye Research Unit (VERU), School of Medicine<br>López-Sánchez, Guillermo Felipe; Faculty of Sport Sciences, University of Murcia<br>Hu, Liang; Zhejiang University, Physical Education<br>Chao, Cao; Division of Public Health Sciences, Department of Surgery, Washington University School of Medicine, St Louis, MO, USA; Centre for Public Health Systems Science, Brown School Washington University, St Louis, MO USA.<br>Vancampfort, D.; Department of Rehabilitation Sciences, KU Leuven, University of Leuven, Leuven, Belgium; University Psychiatric Centre, KU Leuven, University of Leuven, Leuven-Kortenberg, Belgium<br>Koyanagi, Ai; Parc Sanitari Sant Joan de Deu, Research and Development Unit<br>Stubbs, B; King's College London<br>Firth, Joseph ; University of Western Sydney, NICM Health Research Institute<br>Yang, Lin; Department of Epidemiology, Medical University of Vienna, Austria

Keywords:	Physical activity, Sedentary behaviour, Accelerometer, Visual impairment, Diabetic retinopathy < DIABETES & ENDOCRINOLOGY, NHANES

SCHOLARONE™
Manuscripts

Visual impairment and objectively measured physical activity and sedentary behavior in US adolescents and adults

Lee Smith[1*], Sarah E Jackson[2*], Shahina Pardhan[3], Guillermo Felipe López-Sánchez[4], Liang Hu[5], Chao Cao[6], Davy Vancampfort[7], Ai Koyanagi[8], Brendon Stubbs[9], Joseph Firth[10], Lin Yang[11]

1. The Cambridge Centre for Sport and Exercise Sciences, Anglia Ruskin University, Cambridge

2. Department of Behavioural Science and Health, UCL, London, UK

3. Vision & Eye Research Unit (VERU), School of Medicine, Anglia Ruskin University, Cambridge, UK

4. Faculty of Sport Sciences, University of Murcia, Spain.

5. Department of sport science, Zhejiang University, Hangzhou, China

6.Division of Public Health Sciences, Department of Surgery, Washington University School of Medicine, St Louis, MO, USA; Centre for Public Health Systems Science, Brown School Washington University, St Louis, MO USA.

7. Department of Rehabilitation Sciences, KU Leuven, University of Leuven, Leuven, Belgium; University Psychiatric Centre, KU Leuven, University of Leuven, Leuven-Kortenberg, Belgium;

For peer review only - http://bmjopen.bmj.com/site/about/guidelines.xhtml

8. Research and Development Unit, Parc Sanitari Sant Joan de Déu, Universitat de Barcelona, Fundació Sant Joan de Déu, Dr Antoni Pujadas, 42, SantBoi de Llobregat, Barcelona, 08830, Spain; Instituto de Salud Carlos III, Centro de Investigación Biomédicaen Red de Salud Mental, CIBERSAM, Monforte de Lemos 3-5 Pabellón 11, Madrid, 28029, Spain;

9. Physiotherapy Department, South London and Maudsley NHS Foundation Trust, Denmark Hill, London, UK; Health Service and Population Research Department, Institute of Psychiatry, Psychology and Neuroscience, King's College London, De Crespigny Park, London, UK; Faculty of Health, Social Care and Education, Anglia Ruskin University, Chelmsford, UK.

10. NICM, School of Science and Health, University of Western Sydney, Sydney, Australia; Division of Psychology and Mental Health, Faculty of Biology, Medicine and Health, University of Manchester, Manchester, UK.

11. Department of Epidemiology, Medical University of Vienna, Austria

*These authors share first authorship and are corresponding authors:

Lee Smith: lee.smith@anglia.ac.uk Sarah E Jackson: s.e.jackson@ucl.ac.uk

Word count, excluding title page, abstract, references, figures and tables: 2977.

For peer review only

For peer review only - http://bmjopen.bmj.com/site/about/guidelines.xhtml

ABSTRACT

Objectives: The aim of this study was to compare levels of physical activity and sedentary time in a representative sample of US adolescents and adults with and without visual impairment. **Design:** Cross-sectional analyses were carried out using data from the National Health and Nutrition Examination Survey. **Participants:** The study population consisted of 6,001 participants (adolescents n= 1766, adults n= 4235).The present analysis aggregated data from waves 2003-2004 and 2005-2006. **Measures**: Objective physical activity and sedentary behaviour assessment was implemented for 7 days. Distance visual acuity was measured for all participants 12 years and older for each eye. Participants were categorised as: normal vision, uncorrected refractive error, nonrefractive visual impairment. We estimated the sex-specific linear associations between presenting vision and objectively measured physical activity and sedentary patterns using adjusted generalized linear models in adolescents and adults. **Results and conclusions:** Adolescents with uncorrected refractive error and nonrefractive visual impairment did not accumulate higher levels of sedentary time or lower levels of MVPA compared to those with normal vision. We observed no association between vision status and accelerometer measured MVPA in adults aged 20 and older. We observed more time spent sedentary among females with nonrefractive visual impairment compared to those presenting normal vision (mean difference 237.9 mins/week, 95% CI: 23.4 to 456.5). Adults with nonrefractive visual impairment appeared to accumulate lower lifestyle physical activity, particularly in females (mean difference -118.3 mins/week, 95% CI: -168.7 to -67.9). Adult women

For peer review only

For peer review only - http://bmjopen.bmj.com/site/about/guidelines.xhtml

with nonrefractive visual impairment have lower levels of lifestyle physical activity and higher levels of sedentary time than those with normal vision.

Key words: Physical activity, sedentary behaviour, accelerometer, visual impairment, diabetic retinopathy, NHANES.

STRENGTHS AND LIMITATIONS OF THIS STUDY

- Large population based sample of US adolescents and adults.
- Objective measure of physical activity and sedentary time.
- Analyses are of a cross-sectional design and thus it is not known whether visual impairment leads to low levels of activity and high levels of sedentary time or vice versa.
- Adequate levels of physical activity and lower sedentary time may decrease risk for visual impairment by reducing risk for diabetes and associated complications such as cataracts and diabetic retinopathy.

For peer review only - http://bmjopen.bmj.com/site/about/guidelines.xhtml

INTRODUCTION

Physical activity may be defined as any bodily movement caused by contraction of skeletal muscle that results in energy expenditure[1] and may include activities such as structured exercise and sport, active travel (walking and cycling), occupational activity, and household chores/ gardening. Regular and sustained participation in physical activity is beneficial for almost every facet of health in children, adolescents and adults. For example, it has been associated with lower incidence of cardiovascular disease, cancer and osteoarthritis, and promotes positive mental health in all ages.[2] At the other end of the energy expenditure spectrum, excessive time in sedentary behaviour has been shown to be detrimental to both physical and mental health, and this is independent from physical activity levels.[3]

In light of the positive benefits of regular participation in physical activity and the detrimental effects of sedentary behaviour, guidelines have been developed. The World Health Organisation states that to maintain good health adults should achieve at least 30 minutes of moderate activity (e.g. brisk walking) on five or more days of the week.[4] Moreover, country specific guidelines state that sedentary time should be kept to a minimum.[5-8] Whereas, children and adolescents should achieve 60 minutes of moderate activity on each day of the week and keep sedentary time to a minimum.[4] However, despite these recommendations population levels of physical activity are low, particularly in Western countries.[9] Data from The National Health Nutrition and Examinations Survey (NHANES) showed that from 2005-2006 fewer than 10% of US adults meet the recommended physical activity guidelines.[9] It is likely that any increase

For peer review only - http://bmjopen.bmj.com/site/about/guidelines.xhtml

in energy expenditure is beneficial to health. It has been hypothesised that increases in physical activity energy expenditure may have health benefits regardless of how that increase is achieved (i.e., achieved via short periods of moderate activity or long periods of light-intensity activity that may yield equivalent levels of physical activity energy expenditure).[10]Considering these potential health benefits all levels of physical activity should be promoted in all populations.

Persons with disabilities have been shown to have low levels of physical activity and high sedentary behaviour levels and understanding differences versus the general population is important.[11] The disability of reduced eyesight may be a key barrier to an active lifestyle in adults and adolescents. The authors of the present paper have shown that in a sample of 6634UK participants (mean age 65.0±9.2 years) those with low vision were twice as likely to be physically inactive than those with good eyesight. Similar findings were found for the variable 'recognition of friends across street' and 'reading ordinary newspaper'.[12] We have also found similar associations in young people.[13] However, key limitations to these analyses were that crude self-reported measures of vision were used (not allowing one to distinguish between types of eye conditions, refractive and nonrefractive) as well as self-reported physical activity. Self-reported physical activity is subjected to bias owing to participants not being able to accurately recall physical activity and reporting higher levels of physical activity than the actual truth. Further research is needed using objective measures of vision and objective measures of physical activity behaviour such as accelerometers, which are a more accurate measure of free living physical activity. Moreover, the discussed

For peer review only

For peer review only - http://bmjopen.bmj.com/site/about/guidelines.xhtml

analyses should be repeated in other populations of those with reduced eyesight owing to different social and political contexts between countries.

One previous study has looked at visual impairment and objective physical activity using the National Health and Nutrition Examination Survey (NHANES) cohort. This study found that in those older than 20 years individuals with normal sight took an average of 9964 steps per day and engaged in an average of 23.5 minutes per day of moderate-to-vigorous physical activity (MVPA), as compared with 9742 steps per day and 23.1 minutes per day of MVPA in individuals with uncorrected refractive error ($P > .50$ for both) and 5992 steps per day and 9.3 minutes/d of MVPA in individuals with visual impairment ($P < .01$ for both). In multivariable models, individuals with visual impairment took 26% fewer steps per day ($P < .01$; 95% CI, 18%-34%) and spent 48% less time in MVPA ($P < .01$; 95% CI, 37%-57%) than individuals with normal sight. However, this study did not look at associations between sedentary time, light physical activity and visual impairment and did not look at levels of activity in adolescence.[14]

The present paper aims to compare levels of physical activity (light, MVPA, lifestyle) and sedentary time in a representative sample of US adolescents and adults using objective measures of physical activity and visual impairment.

For peer review only - http://bmjopen.bmj.com/site/about/guidelines.xhtml

METHODS

Study Population

The NHANES was designed to provide cross-sectional estimates on the prevalence of health, nutrition, and potential risk factors among the civilian non-institutionalized U.S. population up to 85 years of age.[15] In brief, NHANES surveys a nationally representative complex, stratified, multistage, probability clustered sample of about 5,000 participants each year in 15 counties across the country. Survey participants were asked to attend a physical examination in a mobile examination centre (MEC) or in the participants' home. The present analysis aggregated data from waves 2003-2004 and 2005-2006. During these waves objective physical activity and sedentary behaviour assessment was implemented in the NHANES participants by fitting them with a hip-worn accelerometer (ActiGraph AM-7164) for 7 days. The NHANES obtained ethical approval from the National Centre for Health Statistics Research Ethics Review Board and participants provided written informed consent.

We extracted data on socio-demographic information, measures of adiposity, smoking history, vision examination and objective physical activity and combined them into a single dataset for each data collection wave, 2003-2004 and 2005-2006, and excluded those who were pregnant. We further excluded those with physical functional impairments that limited their ability to crawl, walk, run or play (age 12-19 years old) or limited them from walking for a quarter mile, or walking up ten steps (20 years and above).

For peer review only

For peer review only - http://bmjopen.bmj.com/site/about/guidelines.xhtml

Presenting visual acuity

NHANES participants undertook the vision examination at the MEC. The procedure of vision examination has been detailed elsewhere.[16] In brief, distance visual acuity was measured for all participants 12 years and older for each eye. An autorefractor (ARK0760, Nidek Co Ltd, Tokyo, Japan) was used which contains built-in visual acuity charts with 20/20, 20/25, 20/30, 20/40, 20/50, 20/60, 20/80 and 20/200 lines. Presenting visual acuity was defined using the better eyes with participants' usual distance version correction, if any. The 20/50 line was presented first, with at least 4 of the 5 characters to be read correctly to advance to the next line, otherwise the 20/200 line was presented. For eyes with presenting visual acuity of 20/30 or worse, visual acuity were measured after incorporating information from the objective refraction measurement. Participants with better seeing eyes with distance visual acuity of 20/30 or better were categorized as normal vision. Participants with better-seeing eyes of presenting visual impairment that improved, aided by automated refraction result to 20/40 or better were categorized as uncorrected refractive error, otherwise nonrefractive visual impairment.[16]

Accelerometer measured activity pattern

NHANES participants were asked during their physical examinations at the MEC to wear an accelerometer (ActiGraph AM-7164, 1-minute epochs) on the right hip for 7 consecutive days to objectively measure free-living physical activity. The ActiGraph AM-7164 is a validated, small lightweight device that provides detailed information about the

For peer review only - http://bmjopen.bmj.com/site/about/guidelines.xhtml

intensity, frequency and duration of physical activity.[17] The epoch length was set at 1 minute, and the Actigraph recorded count data for physical activity in the form of counts per minute (cpm). Non-wear time was defined as 60 minutes of consecutive zero counts. A recording of at least 10 hours of data was defined as a valid day, and four or more valid days were required to be included in the analysis. The total minutes of valid data were recorded as the accelerometer wear time. Based on standard cpm cutoff methods[18], four raw activity outcomes were derived: sedentary behaviour (<100 cpm), light intensity physical activity (100-759 cpm), lifestyle activity or ambulatory(760-2109) and at least 10 minutes of moderate-to-vigorous physical activity (>2020 cpm). We further computed wearing time adjusted activity by dividing each raw activity minutes by total wear time and multiplying the resulting fraction by the average wear time of all participants. We summarized the adjusted total weekly minutes of sedentary behaviour, light intensity physical activity, lifestyle physical activity, and moderate-to-vigour physical activity for each participant.

Socio-demographic characteristics

Socio-demographic characteristics including age, sex, race and ethnicity, household income, and smoking status were extracted. Based on self-reported race and ethnicity, participants were classified into one of the three racial groups: Non-Hispanic white, Non-Hispanic black, and Hispanic and others. Annual household income were grouped into <25,000; 25000-44999; 45000-74999; and ≥75,000 US dollars. We classified participants into three groups: never smokers (did not smoke 100 cigarettes and do not

For peer review only - http://bmjopen.bmj.com/site/about/guidelines.xhtml

204 smoke now), former smokers (smoked 100 cigarettes in life and do not smoke now),
205 and current smokers (smoked 100 cigarettes in life and smoke now).

206 **Overweight and obesity criteria**

207 Weight and height were measured during the physical examination in a mobile
208 examination centre or in the participant's home for those whose travels were limited.
209 The measurements followed standard procedures and were carried out by trained
210 technicians with standardized equipment. BMI was calculated as weight in kg / (height
211 in meters)^2. The standard definition for overweight and obesity classification was used
212 to divide the BMI values into three categories: underweight or normal weight (<25.0),
213 overweight (25.0 – 29.9), and obese (≥30.0).

214 **Chronic illness**

215 We included four chronic conditions: cardiovascular diseases, diabetes, cancer and
216 arthritis. Participants were considered as having chronic illness if they self-reported ever
217 been told by a physician that they have the following conditions: congestive heart
218 failure, coronary heart disease, heart attack, a stroke (cardiovascular diseases),
219 diabetes, cancer, or arthritis.

220

221

For peer review only - http://bmjopen.bmj.com/site/about/guidelines.xhtml

222 **Statistical Analysis**

223 Survey analysis procedures were used to account for the sample weights, stratification, and clustering of the complex sampling design to ensure nationally representative estimates. We calculated the descriptive statistics for participant characteristics by presenting vision. We summarized sample size and weighted proportion.

227 We estimated the sex-specific linear associations between presenting vision (normal vision, uncorrected refractive error, nonrefractive visual impairment) and objectively measured physical activity and sedentary patterns using generalized linear model in children and adolescents age between 12-19 years old, and adult 20 years and older, respectively. Generalized linear models were adjusted for age, race, BMI and household income among those between 12-19 years old, and additionally adjusted for smoking and chronic illness among adults 20 years and older. Due to the small amount of light intensity physical activity and lifestyle physical activity in the younger population, we only included sedentary time and moderate to vigorous physical activity in models for population age 12-19 years old.

237 Finally, we estimated the marginal mean in multivariate adjusted generalized linear models for each modelled outcomes of accelerometer measured activity pattern. All statistical significance was set at *p*<0.05.All statistical analyses were performed using Stata version 14.0 (STATA Corp., College Station, Texas, USA).

241

For peer review only - http://bmjopen.bmj.com/site/about/guidelines.xhtml

RESULTS

Our study population consisted of 6,001 participants who had data on presenting refractive error and accelerometer measured activity pattern. The majority of the participants were 20 years and older (n=4235). Overall, 60.8% (weight proportion) of participants had presenting normal vision, 33.8% and 5.4% had uncorrected refractive error or non-refractive visual impairment. Participants who were older, female, with education high school or lower and chronic conditions were more likely to have nonrefractive visual impairment compared to younger, male, well-educated participants and those with no chronic illness conditions. (Table 1).

Tables 2 and 3 summarize both the non-adjusted and adjusted associations between presenting vision status and accelerometer measured activity pattern in younger (12-19 years old) participants and adults who were 20 years and older. Children and adolescents aged between 12-19 years old with uncorrected refractive error and nonrefractive visual impairment did not accumulate higher levels of sedentary time or lower levels of MVPA compared to those with normal vision. The null association was seen in both male and female. After adjusting for age, BMI, race and household income, the estimated marginal mean of sedentary time in those with nonrefractive visual impairment were 3982.7 minutes per week (equivalent 9.5 hours a day) and 3722.9 minutes per week (equivalent 8.8 hours a day) in male and female, respectively. The estimated marginal mean of MVPA in those with nonrefractive visual impairment were 156 minutes per week in male and 55 minutes per week in female.

For peer review only

For peer review only - http://bmjopen.bmj.com/site/about/guidelines.xhtml

Table 1. Socio-demographic characteristics and objectively measured physical activity level of US population 12 and older from the NHNAES (2003-2006) by visual impairment

	Normal vision		Uncorrected refractive error		Visual impairment		
	N	Weighted %	N	Weighted %	N	Weighted %	p
Overall	3350	60.8%	2225	33.8%	426	5.4%	
Weighted N	70084202		38937983		6184612		
Age group, y							0.001
12-19	999	11.2%	689	12.4%	78	6.8%	
≥20	2351	88.8%	1536	87.6%	348	93.2%	
Sex							0.004
Men	1837	53.5%	1114	47.0%	223	49.9%	
Women	1513	46.5%	1111	53.0%	203	50.1%	
Race/ethnicity							0.257
Non-Hispanic white	1555	74.5%	950	71.3%	229	77.0%	
Non-Hispanic black	802	9.6%	543	10.7%	86	9.5%	
Hispanic	993	15.9%	732	18.0%	111	13.5%	
Other	133	4.8%	91	5.7%	8	3.8%	
Education[a]							<.001
<High school	964	17.5%	806	24.7%	209	38.6%	
High school	1740	52.2%	1162	53.5%	218	56.8%	
>High school	976	36.5%	511	31.6%	56	18.0%	
Weight status[b]							0.649
Normal	1416	37.1%	989	39.4%	171	37.5%	
Overweight	1009	32.4%	655	32.3%	141	32.0%	
Obesity	911	30.0%	571	27.9%	112	30.1%	
Smoking status[a]							0.079
Never smoker	1243	46.0%	780	45.0%	167	44.6%	
Former smoker	611	22.7%	450	24.2%	130	33.3%	
Current smoker	496	20.0%	306	18.4%	51	15.3%	
Chronic condition[c]							<.001
Yes	649	23.1%	616	30.3%	180	44.4%	

[a]Data are only available on adults age 20 years and older

For peer review only - http://bmjopen.bmj.com/site/about/guidelines.xhtml

[b]Body mass index was calculated as weight in kilograms divided by height in meters squared. Weight status were defined using BMI-to-age percentile in 12-19 years old, and WHO standard cut-off for adults 20 years and older

[c]If participants have one of the following conditions: cardiovascular disease, diabetes, arthritis, and cancer

Table 2. Accelerometer measured activity pattern (minutes per week) by vision status in the NHNAES (2003-2006) children and adolescence 12-19 years old

	Sedentary time									
	Male					Female				
	Un-adjusted β (95%CI)		MV-adjusted β (95%CI)[a]		Marginal mean	Un-adjusted β (95%CI)		MV-adjusted β (95%CI)[a]		Marginal mean
Normal vision	Ref		Ref		3354.3	Ref		Ref		3541.5
Uncorrected refractive error	-50.9	(-326.4 to224.5)	-101.7	(-362.5 to159.2)	3252.6	-63.3	(-257.0 to130.4)	-41.4	(-226.85 to 144.1)	3500.1
Visual impairment	697.5	(-326.4 to1721.3)	628.5	(-296.6 to1553.5)	3982.7	141.5	(-424.5 to707.5)	181.4	(-347.99 to 710.8)	3722.9
P trend	0.578		0.815			0.844		0.960		
	**Moderate to vigorous physical activity**									
	**Male**					**Female**				
	Un-adjusted β (95%CI)		MV-adjusted β (95%CI)[a]		Marginal mean	Un-adjusted β (95%CI)		MV-adjusted β (95%CI)[a]		Marginal mean
Normal vision	Ref		Ref		104.8	Ref		Ref		47.7
Uncorrected refractive error	-1.4	(-24.2 to21.4)	-9.1	(-30.0 to11.8)	95.7	-4.3	(-19.6 to11.0)	-5.7	(-21.4 to10.1)	42.0
Visual impairment	63.9	(-1.2 to129.1)	51.3	(-16.4 to119.0)	156.1	10.2	(-36.9 to57.2)	7.7	(-36.7 to52.1)	55.4
P trend	0.432		0.870			0.888		0.759		

Multivariate adjusted models included age (continuous), BMI, race and household income

For peer review only - http://bmjopen.bmj.com/site/about/guidelines.xhtml

Table 3. Accelerometer measured activity pattern (minutes per week) by vision status in the NHNAES (2003-2006) adults 20 years and older

Sedentary time

	**Male**					**Female**				
	Un-adjusted β (95%CI)		MV-adjusted β (95%CI)[a]		Marginal mean	Un-adjusted β (95%CI)		MV-adjusted β (95%CI)[a]		Marginal mean
Normal vision	Ref		Ref		3519.6	Ref		Ref		3404.4
Uncorrected refractive error	6.5	(-100.6 to113.6)	-25.7	(-130.7 to79.3)	3493.9	124.9	(-11.6 to261.4)	65.5	(-77.2 to208.2)	3470.0
Visual impairment	371.5	(124.6 to618.3)	230.8	(-38.1 to499.7)	3750.5	357.2	(120.1 to594.4)	239.9	(23.4 to456.5)	3644.3
P trend	0.042		0.379			0.002		0.057		

Light intensity physical activity

	**Male**					**Female**				
	Un-adjusted β (95%CI)		MV-adjusted β (95%CI)[a]		Marginal mean	Un-adjusted β (95%CI)		MV-adjusted β (95%CI)[a]		Marginal mean
Normal vision	Ref		Ref		1855.5	Ref		Ref		1919.3
Uncorrected refractive error	-80.6	(-164.5 to3.2)	-64.8	(-145.6 to16.0)	1790.7	-40.7	(-112.8 to31.3)	-34.9	(-106.8 to37.0)	1884.4
Visual impairment	-74.6	(-217.1 to68.0)	7.3	(-125.7 to140.3)	1862.8	-129.9	(-262.5 to2.8)	-120.7	(-254.4 to13.1)	1798.6
P trend	0.044		0.248			0.043		0.063		

Lifestyle physical activity

	**Male**					**Female**				
	Un-adjusted β (95%CI)		MV-adjusted β (95%CI)[a]		Marginal mean	Un-adjusted β (95%CI)		MV-adjusted β (95%CI)[a]		Marginal mean
Normal vision	Ref		Ref		748.4	Ref		Ref		594.8
Uncorrected refractive error	-78.9	(-127.4 to-30.5)	-44.6	(-86.3 to-2.9)	703.8	-74.8	(-113.4 to-36.1)	-35.8	(-69.8 to-1.7)	559.0
Visual impairment	-191.8	(-301.8 to-81.8)	-63.6	(-161.9 to34.6)	684.7	-232.2	(-281.5 to-182.9)	-118.3	(-168.7 to-67.9)	476.5
P trend	0.000		0.039			0.000		0.001		

Moderate to vigorous physical activity

	**Male**					**Female**				
	Un-adjusted β (95%CI)		MV-adjusted β (95%CI)[a]		Marginal mean	Un-adjusted β (95%CI)		MV-adjusted β (95%CI)[a]		Marginal mean

For peer review only

Normal vision	Ref		Ref		56.0	Ref		Ref		42.3
Uncorrected refractive error	-0.6	(-11.3 to10.1)	3.5	(-8.3 to15.2)	59.4	-3.1	(-11.8 to5.6)	0.1	(-7.1 to7.3)	42.4
Visual impairment	-16.5	(-39.0 to6.1)	-1.2	(-26.1 to23.7)	54.7	-4.9	(-22.9 to13.1)	6.4	(-11.7 to24.5)	48.7
P trend		0.321		0.766			0.387		0.594	

[a] Multivariate adjusted models included age (continuous), BMI, race , household income, smoking status, and chronic illness

For peer review only

Similarly, we observed no association between presenting vision status and accelerometer measured MVPA in adults aged 20 and older, although the multivariate marginal mean of MVPA among those with nonrefractive visual impairment (54.7 minutes per week in male, 48.7 minutes per week in female) appeared lower than that in the young population. In respect to sedentary behaviour, we observed higher level of sedentary time among female with nonrefractive visual impairment compared to those with presenting normal vision (mean difference mins/week 237.9, 95% CI: 23.4 to 456.5). We observed no association between presenting vision and light intently physical activity in either gender. However, adults with nonrefractive visual impairment appeared to accumulate lower lifestyle physical activity, particularly in females (mean difference mins/week -118.3, 95% CI: -168.7 to -67.9).

DISCUSSION

In the present study of a large population based sample of the US we found that those 12-19 years old with uncorrected refractive error and nonrefractive visual impairment had similar levels of activity and sedentary time to those presenting normal vision. Among adults aged 20 years and older we found higher levels of sedentary time among women with nonrefractive visual impairment compared to those with normal vision. Moreover, adults with nonrefractive visual impairment appeared to accumulate lower lifestyle physical activity, particularly in women.

For peer review only - http://bmjopen.bmj.com/site/about/guidelines.xhtml

The finding that visually impaired adolescents (uncorrected refractive error and nonrefractive visual impairment) exhibit little difference in their level of activity and sedentary behavior compare to adolescents with “normal” vision is interesting. To our knowledge just one previous study has compared levels of activity in visually impaired adolescents (a combination of uncorrected refractive error and nonrefractive visual impairment) to those with normal vision and found similar findings. In a sample of 53 adolescents completing the International Physical Activity Questionnaire – Short Form it was found that physical activity levels of visually impaired adolescents and sighted adolescents were similar ($p>0.05$).[19]Findings from the present study add to this work through utilizing a large population based sample and objective measures of physical activity. Moreover, the present study is the first to compare differences in sedentary time between adolescents with impaired vision and normal sight. One plausible reason for these findings is that adolescents with normal vision have very low levels of physical activity.[20] Therefore, minimizing any difference in a reduction of physical activity owing to a disability (e.g. reduced eyesight). Another reason for the lack of association could be owing to physical education. Indeed, all adolescents regardless of visual impairment will be required to partake in physical education and thus acquire similar levels of physical activity during the school day. The present study found that the estimated marginal mean of MVPA in those with nonrefractive visual impairment were low, 156 minutes per week in male and 55 minutes per week in female, and sedentary time high. Interventions are needed to promote physical activity in adolescents with visual impairments.

For peer review only - http://bmjopen.bmj.com/site/about/guidelines.xhtml

The present finding that adults with nonrefractive visual impairment accumulated lower lifestyle physical activity than those with normal vision, specifically in women, supports that of previous research and adds to it through the use of objective physical activity measures. For example, in a recent study of 6,634 older English adults those with fair–poor and good eyesight were significantly more likely to be inactive, categorized by self-report, than those who reported excellent eyesight.[12] Further research is needed to understand why those with reduced eyesight have lower levels of physical activity. One plausible explanation may be fear of going outside, owing to falling or suffering other accidents. This low level of physical activity is of concern as this population may be at an increased risk of chronic illness, such as higher risk of cancer[21] and also have associated risk factors such as higher smoking rates,[22] independent of physical activity. Moreover, those who are visually impaired often report having a low quality of life.[23] Further compounding is a high prevalence of sedentary time in this population. Indeed, for the first time, the present study has shown higher levels of sedentary time among women with nonrefractive visual impairment compared to those with normal vision. Refractory visual problems can normally be corrected and are likely to be less disabling than nonrefractive visual impairment. Moreover, nonrefractive visual impairment are likely to be comorbid. Therefore, those with nonrefractive visual impairment may be a population with greater barriers to physical activity participation. A rationale for stronger associations with higher levels of sedentary behavior and lower levels of physical activity in women but not men with visual impairment is elusive and further qualitative research to explain this finding is required.

For peer review only - http://bmjopen.bmj.com/site/about/guidelines.xhtml

A clear strength of this study is a large population based sample of US adolescents and adults and the objective measure of physical activity and sedentary time. However, the data must be interpreted in light of its limitations. Analyses are of a cross-sectional design and thus it is not known whether visual impairment leads to low levels of activity and high levels of sedentary time or vice versa. Indeed, adequate levels of physical activity and lower sedentary time may decrease risk for visual impairment by reducing risk for diabetes and associated complications such as cataracts and diabetic retinopathy.

In conclusion, findings from the present study suggest generally low levels of physical activity and high levels of sedentary time in adolescents. The activity patterns, however are similar between those who were visually impaired and those with normal vision. Adult women with nonrefractive visual impairment have lower levels of lifestyle physical activity and higher levels of sedentary time than those with normal vision. Taken together these findings suggest that interventions are needed to promote physical activity and reduce sedentary time in adult populations with visual impairment, specifically adult women.

For peer review only

REFERENCES

1. Caspersen CJ, Powell KE, Christenson GM. Physical activity, exercise, and physical fitness: definitions and distinctions for health-related research. *Public Health Rep* 1985;100:126-31.

For peer review only - http://bmjopen.bmj.com/site/about/guidelines.xhtml

2. Warburton DER, Bredin SSD. Health benefits of physical activity: a systematic review of current systematic reviews. *Curr Opin Cardiol* 2017;32:541-556.

3. de Rezende LF, Rodrigues Lopes M, Rey-Lopez JP, et al. Sedentary behavior and health outcomes: an overview of systematic reviews. *PLoS One* 2014;9:e105620.

4. World Health Organization. Global recommendations on physical activity for health. Geneva, Switzerland: World Health Organization 2010.

5. Chief Medical Office (CMO): UK physical activity guidelines. https://www.gov.uk/government/publications/uk-physical-activity-guidelines. Accessed Jun 26 2018., 2011

6. The Department of Health: Australia's Physical Activity and Sedentary Behaviour Guidelines. http://www.health.gov.au/internet/main/publishing.nsf/content/health-pubhlth-strateg-phys-act-guidelines. Accessed Jun 26 2018., 2017

7. Bentley GF, Jago R, Turner KM. Mothers' perceptions of the UK physical activity and sedentary behaviour guidelines for the early years (Start Active, Stay Active): a qualitative study. *BMJ Open* 2015;5:e008383.

8. LeBlanc AG, Berry T, Deshpande S, et al. Knowledge and awareness of Canadian Physical Activity and Sedentary Behaviour Guidelines: a synthesis of existing evidence. Appl *Physiol Nutr Metab* 2015;40:716-24.

9. Tucker JM, Welk GJ, Beyler NK. Physical activity in U.S.: adults compliance with the Physical Activity Guidelines for Americans. *Am J Prev Med* 2011;40:454-61.

10. Smith L, Ekelund U, Hamer M. The potential yield of non-exercise physical activity energy expenditure in public health. *Sports Med* 2015;45:449-52.

For peer review only

11. Rimmer JH, Riley B, Wang E, et al. Physical activity participation among persons with disabilities: barriers and facilitators. *Am J Prev Med* 2004;26:419-25.

12. Smith L, Timmis MA, Pardhan S, et al. Physical inactivity in relation to self-rated eyesight: cross-sectional analysis from the English Longitudinal Study of Ageing. *BMJ Open Ophthalmol* 2017;1:e000046.

13. Williams G, Aggio D, Stubbs B, et al. Physical activity levels in children with sensory problems: Cross-sectional analyses from the Millennium Cohort Study. *Disabil Health J* 2018;11:58-61.

14. Willis JR, Jefferys JL, Vitale S, et al. Visual impairment, uncorrected refractive error, and accelerometer-defined physical activity in the United States. *Arch Ophthalmol* 2012;130:329-35.

15. Centers for Disesae Control and Prevention: National Health and Nutrition Examination Survey. http://www.cdc.gov/nchs/nhanes.htm. Accessed June 21, 2016.,

16. Vitale S, Cotch MF, Sperduto RD. Prevalence of visual impairment in the United States. *Jama* 2006;295:2158-63.

17. Melanson EL, Jr., Freedson PS. Validity of the Computer Science and Applications, Inc. (CSA) activity monitor. *Med Sci Sports Exerc* 1995;27:934-40.

18. Crouter SE, DellaValle DM, Haas JD, et al. Validity of ActiGraph 2-regression model, Matthews cut-points, and NHANES cut-points for assessing free-living physical activity. *J Phys Act Health* 2013;10:504-14.

19. Demirturk F, Kaya M. Physical Education Lessons and Activity Status of Visually Impaired and Sighted Adolescents. *Med Sci Monit* 2015;21:3521-7.

For peer review only

For peer review only - http://bmjopen.bmj.com/site/about/guidelines.xhtml

20. Marques A, Gaspar de Matos M. Adolescents' physical activity trends over the years: a three-cohort study based on the Health Behaviour in School-aged Children (HBSC) Portuguese survey. *BMJ Open* 2014;4:e006012.

21. Pukkala E, Verkasalo PK, Ojamo M, et al. Visual impairment and cancer: a population-based cohort study in Finland. *Cancer Causes Control* 1999;10:13-20.

22. Zhang X, Kahende J, Fan AZ, et al. Smoking and visual impairment among older adults with age-related eye diseases. *Prev Chronic Dis* 2011;8:A84.

23. Anokye NK, Trueman P, Green C, et al. Physical activity and health related quality of life. *BMC Public Health* 2012;12:624.

For peer review only

Authors Contributions: LY did the analyses. LS interpreted the results and drafted the manuscript. All authors critically appraised the manuscript and approved the final version before submission.

Funding Statement: Seneca Foundation—Agency for Science and Technology of the Region of Murcia (Spain). Postdoctoral fellowship: Guillermo Felipe López Sánchez.

Competing Interests Statement: No conflicting relationship exists for any author.

For peer review only - http://bmjopen.bmj.com/site/about/guidelines.xhtml

STROBE 2007 (v4) Statement—Checklist of items that should be included in reports of *cross-sectional studies*

Section/Topic	Item #	Recommendation	Reported on page #
**Title and abstract**	1	(*a*) Indicate the study's design with a commonly used term in the title or the abstract	1
		(*b*) Provide in the abstract an informative and balanced summary of what was done and what was found	3-4
**Introduction**			
Background/rationale	2	Explain the scientific background and rationale for the investigation being reported	5-7
Objectives	3	State specific objectives, including any prespecified hypotheses	7
**Methods**			
Study design	4	Present key elements of study design early in the paper	8
Setting	5	Describe the setting, locations, and relevant dates, including periods of recruitment, exposure, follow-up, and data collection	8
Participants	6	(*a*) Give the eligibility criteria, and the sources and methods of selection of participants	8
Variables	7	Clearly define all outcomes, exposures, predictors, potential confounders, and effect modifiers. Give diagnostic criteria, if applicable	9-11
Data sources/ measurement	8*	For each variable of interest, give sources of data and details of methods of assessment (measurement). Describe comparability of assessment methods if there is more than one group	9-11
Bias	9	Describe any efforts to address potential sources of bias	*8*
Study size	10	Explain how the study size was arrived at	8
Quantitative variables	11	Explain how quantitative variables were handled in the analyses. If applicable, describe which groupings were chosen and why	
Statistical methods	12	(*a*) Describe all statistical methods, including those used to control for confounding	12
		(*b*) Describe any methods used to examine subgroups and interactions	12
		(*c*) Explain how missing data were addressed	12
		(*d*) If applicable, describe analytical methods taking account of sampling strategy	12
		(*e*) Describe any sensitivity analyses	12
**Results**			
Participants	13*	(a) Report numbers of individuals at each stage of study—eg numbers potentially eligible, examined for eligibility,	8

For peer review only

		confirmed eligible, included in the study, completing follow-up, and analysed	
		(b) Give reasons for non-participation at each stage	8
		(c) Consider use of a flow diagram	
Descriptive data	14*	(a) Give characteristics of study participants (eg demographic, clinical, social) and information on exposures and potential confounders	8
		(b) Indicate number of participants with missing data for each variable of interest	8
Outcome data	15*	Report numbers of outcome events or summary measures	13
Main results	16	(*a*) Give unadjusted estimates and, if applicable, confounder-adjusted estimates and their precision (eg, 95% confidence interval). Make clear which confounders were adjusted for and why they were included	13-18
		(*b*) Report category boundaries when continuous variables were categorized	13-18
		(*c*) If relevant, consider translating estimates of relative risk into absolute risk for a meaningful time period	
Other analyses	17	Report other analyses done—eg analyses of subgroups and interactions, and sensitivity analyses	13-18
**Discussion**			
Key results	18	Summarise key results with reference to study objectives	18-21
Limitations	19	Discuss limitations of the study, taking into account sources of potential bias or imprecision. Discuss both direction and magnitude of any potential bias	18-21
Interpretation	20	Give a cautious overall interpretation of results considering objectives, limitations, multiplicity of analyses, results from similar studies, and other relevant evidence	18-21
Generalisability	21	Discuss the generalisability (external validity) of the study results	18-21
**Other information**			
Funding	22	Give the source of funding and the role of the funders for the present study and, if applicable, for the original study on which the present article is based	2

*Give information separately for cases and controls in case-control studies and, if applicable, for exposed and unexposed groups in cohort and cross-sectional studies.

Note: An Explanation and Elaboration article discusses each checklist item and gives methodological background and published examples of transparent reporting. The STROBE checklist is best used in conjunction with this article (freely available on the Web sites of PLoS Medicine at http://www.plosmedicine.org/, Annals of Internal Medicine at http://www.annals.org/, and Epidemiology at http://www.epidem.com/). Information on the STROBE Initiative is available at www.strobe-statement.org.

For peer review only

BMJ Open

Visual impairment and objectively measured physical activity and sedentary behavior in US adolescents and adults: a cross-sectional study

Journal:	*BMJ Open*
Manuscript ID	bmjopen-2018-027267.R1
Article Type:	Research
Date Submitted by the Author:	11-Jan-2019
Complete List of Authors:	Smith, Lee; Anglia Ruskin University - Cambridge Campus, Department of Life Sciences<br>Jackson, Sarah ; University College London, Epidemiology and Public Health;<br>Pardhan, Shahina ; Anglia Ruskin University, Vision and Eye Research Unit (VERU), School of Medicine<br>López-Sánchez, Guillermo Felipe; Faculty of Sport Sciences, University of Murcia<br>Hu, Liang; Zhejiang University, Physical Education<br>Chao, Cao; Division of Public Health Sciences, Department of Surgery, Washington University School of Medicine, St Louis, MO, USA; Centre for Public Health Systems Science, Brown School Washington University, St Louis, MO USA.<br>Vancampfort, D.; Department of Rehabilitation Sciences, KU Leuven, University of Leuven, Leuven, Belgium; University Psychiatric Centre, KU Leuven, University of Leuven, Leuven-Kortenberg, Belgium<br>Koyanagi, Ai; Parc Sanitari Sant Joan de Deu, Research and Development Unit<br>Stubbs, B; King's College London<br>Firth, Joseph ; University of Western Sydney, NICM Health Research Institute<br>Yang, Lin; Department of Epidemiology, Medical University of Vienna, Austria
**Primary Subject Heading**:	Epidemiology
Secondary Subject Heading:	Epidemiology
Keywords:	Physical activity, Sedentary behaviour, Accelerometer, Visual impairment, Diabetic retinopathy < DIABETES & ENDOCRINOLOGY, NHANES

SCHOLARONE™
Manuscripts

Visual impairment and objectively measured physical activity and sedentary behavior in US adolescents and adults: a cross-sectional study

Lee Smith[1]*, Sarah E Jackson[2]*, Shahina Pardhan[3], Guillermo Felipe López-Sánchez[4], Liang Hu[5], Chao Cao[6], Davy Vancampfort[7], Ai Koyanagi[8], Brendon Stubbs[9], Joseph Firth[10], Lin Yang[11]

1. The Cambridge Centre for Sport and Exercise Sciences, Anglia Ruskin University, Cambridge

2. Department of Behavioural Science and Health, UCL, London, UK

3. Vision & Eye Research Unit (VERU), School of Medicine, Anglia Ruskin University, Cambridge, UK

4. Faculty of Sport Sciences, University of Murcia, Spain.

5. Department of sport science, Zhejiang University, Hangzhou, China

6.Division of Public Health Sciences, Department of Surgery, Washington University School of Medicine, St Louis, MO, USA; Centre for Public Health Systems Science, Brown School Washington University, St Louis, MO USA.

7. Department of Rehabilitation Sciences, KU Leuven, University of Leuven, Leuven, Belgium; University Psychiatric Centre, KU Leuven, University of Leuven, Leuven-Kortenberg, Belgium;

For peer review only

8. Research and Development Unit, ParcSanitariSant Joan de Déu, Universitat de Barcelona, FundacióSant Joan de Déu, Dr Antoni Pujadas, 42, SantBoi de Llobregat, Barcelona, 08830, Spain;Instituto de Salud Carlos III, Centro de InvestigaciónBiomédicaen Red de Salud Mental, CIBERSAM, Monforte de Lemos 3-5 Pabellón 11, Madrid, 28029, Spain;

9. Physiotherapy Department, South London and Maudsley NHS Foundation Trust, Denmark Hill, London, UK; Health Service and Population Research Department, Institute of Psychiatry, Psychology and Neuroscience, King's College London, De Crespigny Park, London, UK; Faculty of Health, Social Care and Education, Anglia Ruskin University, Chelmsford, UK.

10. NICM, School of Science and Health, University of Western Sydney, Sydney, Australia; Division of Psychology and Mental Health, Faculty of Biology, Medicine and Health, University of Manchester, Manchester, UK.

11. Department of Epidemiology, Medical University of Vienna, Austria

*These authors share first authorship and are corresponding authors:

Lee Smith: lee.smith@anglia.ac.uk Sarah E Jackson: s.e.jackson@ucl.ac.uk

Word count, excluding title page, abstract, references, figures and tables: 2977.

For peer review only - http://bmjopen.bmj.com/site/about/guidelines.xhtml

ABSTRACT

Objectives: To compare levels of physical activity and sedentary time in a representative sample of US adolescents and adults with and without visual impairment. **Design:** Cross-sectional analyses were carried out using data from the National Health and Nutrition Examination Survey. **Participants:** The study population consisted of 6,001 participants (adolescents n=1,766, adults n=4,235).The present analysis aggregated data from 2003-2004 and 2005-2006. **Measures**: Objective physical activity and sedentary behaviour assessment was conducted over 7 days. Distance visual acuity was measured for each eye in all participants 12 years and older. Participants' vision was categorised as: normal vision, uncorrected refractive error, nonrefractive visual impairment. We estimated the sex-specific linear associations between presenting vision and objectively measured physical activity and sedentary patterns using adjusted generalized linear models in adolescents and adults. **Results and conclusions:** Adolescents with uncorrected refractive error and nonrefractive visual impairment did not accumulate higher levels of sedentary time or lower levels of MVPA compared with those with normal vision. We observed no association between vision status and accelerometer measured MVPA in adults aged 20-49 years. We observed more time spent sedentary among females 20-49 years old with nonrefractive visual impairment compared with those presenting normal vision (mean difference 329.8 mins/week, 95% CI: 12.5 to 647.0). Adults 50 years and older with nonrefractive visual impairment appeared to accumulate less lifestyle physical activity, particularly in women (mean difference -82.8 mins/week, 95% CI: -147.8 to -17.8).Adult women with nonrefractive visual impairment have lower levels of lifestyle physical activity and higher

For peer review only - http://bmjopen.bmj.com/site/about/guidelines.xhtml

levels of sedentary time than those with normal vision. Taken together, these findings highlight the need for interventions to promote physical activity and reduce sedentary time in adult populations with visual impairment, specifically adult women.

Key words: Physical activity, sedentary behaviour, accelerometer, visual impairment, diabetic retinopathy, NHANES.

STRENGTHS AND LIMITATIONS OF THIS STUDY

- Large, population-based sample of US adolescents and adults.
- Objective measures of physical activity and sedentary time.
- Analyses are of a cross-sectional design and thus it is not known whether visual impairment leads to low levels of activity and high levels of sedentary time or vice versa.
- Risk for developing diabetes and associated complications such as cataracts and diabetic retinopathy may be reduced in those with visual impairments by participating in adequate levels of physical activity and lower amounts of sedentary behavior.

For peer review only - http://bmjopen.bmj.com/site/about/guidelines.xhtml
For peer review only

INTRODUCTION

Physical activity may be defined as any bodily movement caused by contraction of skeletal muscle that results in energy expenditure[1] and may include activities such as structured exercise and sport, active travel (walking and cycling), occupational activity, and household chores/gardening. Regular and sustained participation in physical activity is beneficial for almost every facet of health in children, adolescents and adults. For example, it has been associated with lower incidence of cardiovascular disease, cancer and osteoarthritis, and promotes positive mental health in all ages.[2] At the other end of the energy expenditure spectrum, excessive time in sedentary behaviour has been shown to be detrimental to both physical and mental health, and this is independent from physical activity levels.[3]

In light of the positive benefits of regular participation in physical activity and the detrimental effects of sedentary behaviour, guidelines have been developed. The World Health Organisation states that to maintain good health, adults should achieve at least 30 minutes of moderate activity (e.g. brisk walking) on five or more days of the week.[4] Moreover, country-specific guidelines state that sedentary time should be kept to a minimum.[5-8] It is advised that children and adolescents should achieve 60 minutes of moderate activity on each day of the week and keep sedentary time to a minimum.[4] However, despite these recommendations population levels of physical activity are low, particularly in Western countries.[9] Data from the National Health Nutrition and Examinations Survey (NHANES) showed that in 2005-2006 fewer than 10% of US adults met the recommended physical activity guidelines.[9] It is likely that any increase in

For peer review only - http://bmjopen.bmj.com/site/about/guidelines.xhtml

energy expenditure is beneficial to health. It has been hypothesised that increases in physical activity energy expenditure may have health benefits regardless of how that increase is achieved (i.e., achieved via short periods of moderate activity or long periods of light-intensity activity that may yield equivalent levels of energy expenditure).[10] Considering these potential health benefits, all levels of physical activity should be promoted in all populations.

Persons with disabilities have been shown to have low levels of physical activity and high levels of sedentary behaviour and understanding differences versus the general population is important.[11] The disability of reduced eyesight may be a key barrier to an active lifestyle in adults and adolescents. It has been suggested that in people with visual impairment there is a lack of access to recreational and athletic programmes, and help or encouragement in developing suitable and safe physical recreation skills and habits.[12] Moreover, this population experiences activity limitations in walking, and environmental barriers such as transport and lack of accessible exercise equipment can hamper a person's ability to be physically active. [12-13] The authors of the present paper have shown in a sample of 6634 United Kingdom participants (mean age 65.0±9.2 years) those with poor vision were twice as likely to be physically inactive than those with good eyesight. Similar findings were found for the variable 'recognition of friends across street' and 'reading ordinary newspaper'.[14] The present authors have also found similar associations in young people.[15] These findings are of importance owing to a high prevalence of reduced eyesight. For example, in the USA it has been estimated that approximately 14 million individuals aged 12 years or older have visual impairment (defined as distance visual acuity of 20/50 or worse).[16] However, key limitations to

For peer review only - http://bmjopen.bmj.com/site/about/guidelines.xhtml

previously discussed analyses were that crude self-reported measures of vision were used (not allowing one to distinguish between types of eye conditions, refractive and nonrefractive) and physical activity was self-reported. Self-reported physical activity is subject to bias owing to participants not being able to accurately recall physical activity and reporting higher levels of physical activity than the actual truth. Further research is needed using objective measures of vision and objective measures of physical activity behaviour such as accelerometers, which are a more accurate measure of free living physical activity. [17]

One previous study has looked at visual impairment and objective physical activity using the NHANES cohort. This study found that in those older than 20 years, individuals with normal sight took an average of 9964 steps per day and engaged in an average of 23.5 minutes per day of moderate-to-vigorous physical activity (MVPA), as compared with 9742 steps per day and 23.1 minutes per day of MVPA in individuals with uncorrected refractive error (P > .50 for both) and 5992 steps per day and 9.3 minutes/d of MVPA in individuals with visual impairment (P < .01 for both). In multivariable models, individuals with visual impairment took 26% fewer steps per day (P < .01; 95% CI, 18%-34%) and spent 48% less time in MVPA (P < .01; 95% CI, 37%-57%) than individuals with normal sight. However, this study did not look at associations between sedentary time, light physical activity and visual impairment, and did not explore levels of activity in adolescence or older adults.[18]

For peer review only - http://bmjopen.bmj.com/site/about/guidelines.xhtml

The present paper therefore aimed to compare levels of physical activity (light, MVPA, lifestyle) and sedentary time in a representative sample of US adolescents, adults, and older adults using objective measures of physical activity and visual impairment.

For peer review only

For peer review only - http://bmjopen.bmj.com/site/about/guidelines.xhtml

METHODS

Study Population

The NHANES was designed to provide cross-sectional estimates of the prevalence of health, nutrition, and potential risk factors among the civilian non-institutionalized U.S. population up to 85 years of age.[19] In brief, NHANES surveys a nationally representative complex, stratified, multistage, probability clustered sample of around 5,000 participants each year in 15 counties across the country. Survey participants are asked to attend a physical examination either in a mobile examination centre (MEC) or in the participants' home. The present analysis aggregated data from 2003-2004 and 2005-2006. During these waves, objective physical activity and sedentary behaviour assessment was implemented in the NHANES participants by fitting them with a hip-worn accelerometer (ActiGraph AM-7164) for 7 days. The NHANES obtained ethical approval from the National Centre for Health Statistics Research Ethics Review Board and participants provided written informed consent.

Data on socio-demographic information, measures of adiposity, smoking history, vision examination and objective physical activity were extracted and combined into a single dataset for each data collection wave, 2003-2004 and 2005-2006. Further, participants who were pregnant or had physical functional impairments that limited their ability to crawl, walk, run or play (age 12-19 years old) or limited them from walking for a quarter mile, or walking up ten steps (20 years and above) were excluded.

For peer review only - http://bmjopen.bmj.com/site/about/guidelines.xhtml

Presenting visual acuity

NHANES participants undertook the vision examination at the MEC. The procedure of vision examination has been detailed elsewhere.[20] In brief, distance visual acuity was measured for all participants 12 years and older for each eye. An autorefractor (ARK0760, Nidek Co Ltd, Tokyo, Japan) was used which contains built-in visual acuity charts with 20/20, 20/25, 20/30, 20/40, 20/50, 20/60, 20/80 and 20/200 lines. Presenting visual acuity was defined using the better eyes with participants' usual distance version correction, if any. The 20/50 line was presented first, with at least 4 of the 5 characters to be read correctly to advance to the next line, otherwise the 20/200 line was presented. For eyes with presenting visual acuity of 20/30 or worse, visual acuity were measured after incorporating information from the objective refraction measurement. Participants with better seeing eyes with distance visual acuity of 20/30 or better were categorized as having normal vision. Participants with better-seeing eyes of presenting visual impairment that improved, aided by automated refraction result to 20/40 or better were categorized as having uncorrected refractive error, otherwise nonrefractive visual impairment.[20]

Accelerometer measured activity pattern

NHANES participants were asked during their physical examinations at the MEC to wear an accelerometer (ActiGraph AM-7164, 1-minute epochs) on the right hip for 7 consecutive days to objectively measure free-living physical activity. The ActiGraph AM-7164 is a validated, small lightweight device that provides detailed information about the intensity, frequency and duration of physical activity.[21] The epoch length was set at 1

For peer review only

For peer review only - http://bmjopen.bmj.com/site/about/guidelines.xhtml

minute, and the Actigraph recorded data for physical activity in the form of counts per minute (cpm). Non-wear time was defined as 60 minutes of consecutive zero counts. A recording of at least 10 hours of data was defined as a valid day, and four or more valid days were required to be included in the analysis. The total minutes of valid data were recorded as the accelerometer wear time. Based on standard cpm cutoff methods[22], four raw activity outcomes were derived: sedentary behaviour (<100 cpm), light intensity physical activity (100-759 cpm), lifestyle activity or ambulatory (760-2109) and at least 10 minutes of moderate-to-vigorous physical activity (>2020 cpm). We further computed wearing time adjusted activity by dividing each raw activity minutes by total wear time and multiplying the resulting fraction by the average wear time of all participants. We summarized the adjusted total weekly minutes of sedentary behaviour, light intensity physical activity, lifestyle physical activity, and MVPA for each participant.

Socio-demographic characteristics

Socio-demographic characteristics including age, sex, race and ethnicity, household income, employment status, and smoking status were extracted. Based on self-reported race and ethnicity, participants were classified into one of the three racial groups: Non-Hispanic white, Non-Hispanic black, and Hispanic and others. Annual household income was grouped into <20,000; 20,000-74999; and ≥75,000 US dollars. Employment status was dichotomized to employed vs. unemployed. We classified participants into three groups: never smokers (did not smoke 100 cigarettes and do not smoke now), former smokers (smoked 100 cigarettes in life and do not smoke now), and current smokers (smoked 100 cigarettes in life and smoke now).

For peer review only

For peer review only - http://bmjopen.bmj.com/site/about/guidelines.xhtml

216 **Overweight and obesity criteria**

217 Weight and height were measured during the physical examination in MEC or in the
218 participant's home for those whose travel was limited. The measurements followed
219 standard procedures and were carried out by trained technicians with standardized
220 equipment. BMI was calculated as weight in kg / (height in meters)^2. The standard
221 definition for overweight and obesity classification was used to divide the BMI values
222 into three categories: underweight or normal weight (<25.0), overweight (25.0 – 29.9),
223 and obese (≥30.0).

224 **Chronic illness**

225 We included four chronic conditions: cardiovascular diseases, diabetes, cancer and
226 arthritis. Participants were considered as having chronic illness if they self-reported
227 being told by a physician that they have the following conditions: congestive heart
228 failure, coronary heart disease, heart attack, a stroke (cardiovascular diseases),
229 diabetes, cancer, or arthritis.

230 **Patient and Public Involvement**

231 Patients and the public were not involved in the design of the present study.

232

For peer review only - http://bmjopen.bmj.com/site/about/guidelines.xhtml

Statistical Analysis

Survey analysis procedures were used to account for the sample weights, stratification, and clustering of the complex sampling design to ensure nationally representative estimates. Descriptive statistics for participant characteristics were calculated by presenting vision. Sample size and weighted proportions were summarized.

Sex-specific linear associations between presenting vision (normal vision, uncorrected refractive error, nonrefractive visual impairment) and objectively measured physical activity and sedentary patterns were estimated using generalized linear models in children and adolescents aged between 12-19 years old, adult 20-49 years and older adults aged 50+ years, respectively. Generalized linear models were adjusted for age, race, BMI and household income among those between 12-19 years old, and additionally adjusted for employment status, smoking, and chronic illness among adults (20-49 years, and 50 years and older). Due to the small amount of light intensity physical activity and lifestyle physical activity in the younger population, we only included sedentary time and MVPA in models for population age 12-19 years old.

Finally, marginal means were estimated in multivariate adjusted generalized linear models for each modelled outcomes of accelerometer measured activity pattern. All statistical significance was set at $p<0.05$.All statistical analyses were performed using Stata version 14.0 (STATA Corp., College Station, Texas, USA).

For peer review only

RESULTS

The study population consisted of 6,001 participants who had data on presenting refractive error and accelerometer measured activity pattern. The majority of the participants were 20 years and older (n=4235). Overall, 60.8% (weighted proportion) of participants had presenting normal vision, 33.8% and 5.4% had uncorrected refractive error or non-refractive visual impairment. Participants who were older, female, unemployed, with education high school or lower and chronic conditions were more likely to have nonrefractive visual impairment compared with younger, male, employed, well-educated participants and those with no chronic illness conditions. (Table 1).

Tables 2 - 4 summarize both the non-adjusted and adjusted associations between presenting vision status and accelerometer measured activity pattern in younger (12-19 years old) participants and adults aged 20-49 years and ≥50 years, respectively. Children and adolescents aged between 12-19 years old with uncorrected refractive error and nonrefractive visual impairment did not accumulate higher levels of sedentary time or lower levels of MVPA compared with those with normal vision. The null association was seen in both male and female. After adjusting for age, BMI, race and household income, the estimated marginal mean of sedentary time in those with nonrefractive visual impairment were 3982.7 minutes per week (equivalent 9.5 hours a day) and 3722.9 minutes per week (equivalent 8.8 hours a day) in male and female, respectively. The estimated marginal mean of MVPA in those with nonrefractive visual impairment were 156 minutes per week in male and 55 minutes per week in female.

For peer review only - http://bmjopen.bmj.com/site/about/guidelines.xhtml

Table 1. Socio-demographic characteristics and objectively measured physical activity level of US population aged 12 and older from the NHANES (2003-2006) by visual impairment

	Normal vision		Uncorrected refractive error		Visual impairment		
	N	Weighted %	N	Weighted %	N	Weighted %	p
Overall	3350	60.8%	2225	33.8%	426	5.4%	
Weighted N	70084202		38937983		6184612		
Age group, y							0.001
12-19	999	11.2%	689	12.4%	78	6.8%	
20 - 49	1394	57.0%	620	42.1%	58	21.9%	
≥50	937	31.8%	916	44.5%	290	71.3%	
Sex							0.004
Men	1837	53.5%	1114	47.0%	223	49.9%	
Women	1513	46.5%	1111	53.0%	203	50.1%	
Race/ethnicity							0.257
Non-Hispanic white	1555	74.5%	950	71.3%	229	77.0%	
Non-Hispanic black	802	9.6%	543	10.7%	86	9.5%	
Hispanic	993	15.9%	732	18.0%	111	13.5%	
Other	133	4.8%	91	5.7%	8	3.8%	
Household income							<.001
< 20,000	964	17.5%	806	24.7%	209	38.6%	
20000-74999	1740	52.2%	1162	53.5%	218	56.8%	
≥75,000	976	36.5%	511	31.6%	56	18.0%	
Weight status[a]							0.649
Normal	1416	37.1%	989	39.4%	171	37.5%	
Overweight	1009	32.4%	655	32.3%	141	32.0%	
Obesity	911	30.0%	571	27.9%	112	30.1%	
Employment status[b]							
Unemployed	664	23.0%	724	35.9%	242	59.0%	
Employed	1604	77.0%	780	64.1%	98	41.0%	
Smoking status[b]							0.079
Never smoker	1243	46.0%	780	45.0%	167	44.6%	

For peer review only - http://bmjopen.bmj.com/site/about/guidelines.xhtml

Former smoker	611	22.7%	450	24.2%	130	33.3%	
Current smoker	496	20.0%	306	18.4%	51	15.3%	
Chronic condition[c]							<.001
Yes	649	23.1%	616	30.3%	180	44.4%	

[a]Body mass index was calculated as weight in kilograms divided by height in meters squared. Weight status were defined using BMI-to-age percentile in 12-19 years old, and WHO standard cut-off for adults 20 years and older

[b]Data are only available on adults age 20 years and older

[c]If participants have one of the following conditions: cardiovascular disease, diabetes, arthritis, and cancer

For peer review only

Table 2. Accelerometer measured activity pattern (minutes per week) by vision status in the NHANES (2003-2006) children and adolescentsaged 12-19 years

	Sedentary time									
	Male					Female				
	Un-adjusted β (95%CI)		MV-adjusted β (95%CI)[a]		Marginal mean	Un-adjusted β (95%CI)		MV-adjusted β (95%CI)[a]		Marginal mean
Normal vision	Ref		Ref		3354.3	Ref		Ref		3541.5
Uncorrected refractive error	-50.9	(-326.4 to224.5)	-101.7	(-362.5 to159.2)	3252.6	-63.3	(-257.0 to130.4)	-41.4	(-226.85 to 144.1)	3500.1
Visual impairment	697.5	(-326.4 to1721.3)	628.5	(-296.6 to1553.5)	3982.7	141.5	(-424.5 to707.5)	181.4	(-347.99 to 710.8)	3722.9
P trend	0.578		0.815			0.844		0.960		
	**Moderate to vigorous physical activity**									
	**Male**					**Female**				
	Un-adjusted β (95%CI)		MV-adjusted β (95%CI)[a]		Marginal mean	Un-adjusted β (95%CI)		MV-adjusted β (95%CI)[a]		Marginal mean
Normal vision	Ref		Ref		104.8	Ref		Ref		47.7
Uncorrected refractive error	-1.4	(-24.2 to21.4)	-9.1	(-30.0 to11.8)	95.7	-4.3	(-19.6 to11.0)	-5.7	(-21.4 to10.1)	42.0
Visual impairment	63.9	(-1.2 to129.1)	51.3	(-16.4 to119.0)	156.1	10.2	(-36.9 to57.2)	7.7	(-36.7 to52.1)	55.4
P trend	0.432		0.870			0.888		0.759		

[a] Multivariate adjusted models included age (continuous), BMI, race and household income

For peer review only

For peer review only - http://bmjopen.bmj.com/site/about/guidelines.xhtml

Table 3. Accelerometer measured activity pattern (minutes per week) by vision status in the NHANES (2003-2006) adults aged 20 -49 years

	Male					Female				
	Un-adjusted β (95%CI)		MV-adjusted β (95%CI)[a]		Marginal mean	Un-adjusted β (95%CI)		MV-adjusted β (95%CI)[a]		Marginal mean
**Sedentary time**										
Normal vision	Ref		Ref		3367.7	Ref		Ref		3326.2
Uncorrected refractive error	-55.8	(-213.0 to 101.4)	-13.5	(-180.8 to 153.9)	3354.2	-5.0	(-184.5 to 174.5)	9.9	(-193.6 to 213.4)	3336.1
Visual impairment	52.7	(-496.7 to 602.2)	273.9	(-323.3 to 871.0)	3641.5	257.9	(-90.8 to 606.5)	329.8	(12.5 to 647.0)	3655.9
P trend	0.646		0.659			0.666		0.500		
**Light intensity physical activity**										
Normal vision	Ref		Ref		1871.5	Ref		Ref		1900.1
Uncorrected refractive error	41.3	(-71.8 to 154.3)	25.1	(-86.5 to 136.8)	1896.6	23.7	(-84.1 to 131.5)	6.5	(-101.6 to 114.7)	1906.7
Visual impairment	292.8	(-37.5 to 623.1)	7.3	(-77.9 to 505.1)	2085.1	-5.7	(-256.4 to245.0)	-15.5	(-264.5 to 233.4)	1884.6
P trend	0.047		0.198			0.717		0.953		
**Lifestyle physical activity**										
Normal vision	Ref		Ref		815.9	Ref		Ref		654.5
Uncorrected refractive error	9.9	(-55.74 to 75.5)	-2.4	(-70.1 to 65.3)	813.5	-13.3	(-59.6 to 33.1)	-15.0	(-60.6 to 30.6)	639.5
Visual impairment	115.9	(-112.8 to 344.5)	45.9	(-157.2 to 249.1)	861.8	-68.6	(-187.6 to- 50.3)	-81.3	(-181.7 to 19.0)	573.2
P trend	0.330		0.842			0.337		0.249		

Moderate to vigorous physical activity

	Male					Female				
	Un-adjusted β (95%CI)		MV-adjusted β (95%CI)[a]		Marginal mean	Un-adjusted β (95%CI)		MV-adjusted β (95%CI)[a]		Marginal mean

For peer review only - http://bmjopen.bmj.com/site/about/guidelines.xhtml

Normal vision	Ref		Ref		62.6	Ref		Ref		49.8
Uncorrected refractive error	11.4	(-4.2 to 27.0)	10.2	(-6.8 to 27.2)	72.9	2.1	(-8.4 to 12.6)	-2.9	(-11.5 to 5.7)	46.9
Visual impairment	2.7	(-28.3 to 33.7)	3.9	(-25.9 to 33.7)	66.5	1.7	(-29.3 to 32.7)	-2.7	(-25.5 to 20.1)	47.1
P trend		0.226		0.287			0.624		0.457	

Multivariate adjusted models included age (continuous), BMI, race , household income, employment status, smoking status, and chronic illness

For peer review only

For peer review only - http://bmjopen.bmj.com/site/about/guidelines.xhtml

Table 4. Accelerometer measured activity pattern (minutes per week) by vision status in the NHANES (2003-2006) adults aged 50 years and older

	Male					Female				
**Sedentary time**										
	Un-adjusted β (95%CI)		MV-adjusted β (95%CI)[a]		Marginal mean	Un-adjusted β (95%CI)		MV-adjusted β (95%CI)[a]		Marginal mean
Normal vision	Ref		Ref		3756.2	Ref		Ref		3502.5
Uncorrected refractive error	-42.0	(-196.9 to 112.9)	-100.7	(-271.5 to 70.0)	3655.5	211.0	(47.9 to 374.2)	123.8	(-52.4 to 300.0)	3626.3
Visual impairment	290.9	(-26.9 to 608.7)	230.8	(-183.3 to 513.0)	3921.0	318.3	(44.4 to 592.2)	179.6	(-104.9 to 464.1)	3682.1
P trend	0.244		0.816			0.003		0.098		
**Light intensity physical activity**										
	Un-adjusted β (95%CI)		MV-adjusted β (95%CI)[a]		Marginal mean	Un-adjusted β (95%CI)		MV-adjusted β (95%CI)[a]		Marginal mean
Normal vision	Ref		Ref		1815.6	Ref		Ref		1914.4
Uncorrected refractive error	-202.7	(-330.4 to -75.0)	-134.9	(-257.6 to -12.2)	1680.7	-115.9	(-187.5 to -44.3)	-34.9	(-103.6 to 24.8)	1875.0
Visual impairment	-196.0	(-311.91 to -80.1)	-57.2	(-182.2 to 67.81)	1758.4	-194.8	(-335.3 to -54.2)	-56.6	(-205.9 to 92.7)	1857.8
P trend	< .001		0.063			0.001		0.234		
**Lifestyle physical activity**										
	Un-adjusted β (95%CI)		MV-adjusted β (95%CI)[a]		Marginal mean	Un-adjusted β (95%CI)		MV-adjusted β (95%CI)[a]		Marginal mean
Normal vision	Ref		Ref		632.2	Ref		Ref		507.2
Uncorrected refractive error	-126.6	(-190.4 to -62.8)	-54.4	(-106.1 to -2.7)	577.8	-103.5	(-143.2 to -63.8)	-41.0	(-79.8 to -2.3)	466.1
Visual impairment	-224.1	(-298.9 to -149.2)	-56.5	(-140.0 to 27.0)	575.6	-221.0	(-269.2 to -172.8)	-82.8	(-147.8 to -17.8)	424.4
P trend	< .001		0.051			< .001		0.009		
**Moderate to vigorous physical activity**										
	Un-adjusted β (95%CI)		MV-adjusted β (95%CI)[a]		Marginal mean	Un-adjusted β (95%CI)		MV-adjusted β (95%CI)[a]		Marginal mean

For peer review only - http://bmjopen.bmj.com/site/about/guidelines.xhtml

Normal vision	Ref		Ref		48.1	Ref		Ref		30.3
Uncorrected refractive error	-9.2	(-23.3 to 4.9)	-5.0	(-21.5 to 9.4)	42.1	-4.4	(-17.1 to 8.45)	3.4	(-8.6 to 15.4)	33.7
Visual impairment	-16.7	(-43.7 to 10.3)	-5.0	(-36.6 to 26.7)	43.2	0.2	(-21.8 to 22.3)	13.9	(-10.6 to 38.5)	44.2
P trend		0.127		0.579			0.762		0.299	

[a] Multivariate adjusted models included age (continuous), BMI, race , household income, employment status, smoking status, and chronic illness

For peer review only

285 Similarly, no association was observed between presenting vision status and
286 accelerometer measured MVPA in adults aged 20-49 years or those aged ≥50 years,
287 although the multivariate marginal mean of MVPA among those aged ≥50 years with
288 nonrefractive visual impairment (48.1 minutes per week in male, 30.3 minutes per week
289 in female) appeared lower than that in the young adult population (62.6 minutes per
290 week in male, 49.8 minutes per week in female). With respect to sedentary behaviour,
291 higher level of sedentary time was observed among women 20-49 years with
292 nonrefractive visual impairment compared with those with presenting normal vision
293 (mean difference mins/week 329.8, 95% CI: 12.5 to 647.0). No association was
294 observed between presenting vision and light intently physical activity in either gender.
295 However, adults 50 years and older with nonrefractive visual impairment appeared to
296 accumulate lower lifestyle physical activity, particularly in women (mean difference
297 mins/week -82.8, 95% CI: -147.8 to -17.8).

298

299 **DISCUSSION**

300 In the present study of a large population based sample of the US we found that those
301 12-19 years old with uncorrected refractive error and nonrefractive visual impairment
302 had similar levels of activity and sedentary time to those presenting with normal vision.
303 Among adults aged 20-49 years we found higher levels of sedentary time among
304 women with nonrefractive visual impairment compared with those with normal vision.
305 Moreover, adults aged 50 years and older with nonrefractive visual impairment
306 appeared to accumulate lower lifestyle physical activity, particularly in women.

For peer review only - http://bmjopen.bmj.com/site/about/guidelines.xhtml

The finding that visually impaired adolescents (uncorrected refractive error and nonrefractive visual impairment) exhibit little difference in their level of activity and sedentary behavior compared with adolescents with “normal” vision is interesting. To our knowledge, just one previous study has compared levels of activity in visually impaired adolescents (a combination of uncorrected refractive error and nonrefractive visual impairment) with those with normal vision, and it reported similar findings. In a sample of 53 adolescents completing the International Physical Activity Questionnaire – Short Form, physical activity levels of visually impaired adolescents and sighted adolescents were similar ($p>0.05$).[23] Findings from the present study add to this work through utilizing a large, population-based sample and objective measures of physical activity. Moreover, the present study is the first to compare differences in sedentary time between adolescents with impaired vision and normal sight. One plausible reason for these findings is that adolescents with normal vision have very low levels of physical activity;[24] therefore minimizing any difference in a reduction of physical activity owing to a disability (e.g. reduced eyesight). Another reason for the lack of association could be owing to physical education. Indeed, all adolescents regardless of visual impairment are required to partake in physical education and thus acquire similar levels of physical activity during the school day. The present study found that the estimated marginal mean of MVPA in those with nonrefractive visual impairment was low, 156 minutes per week in males and 55 minutes per week in females, and sedentary time high. Interventions are needed to promote physical activity in adolescents with visual impairments.

For peer review only

For peer review only - http://bmjopen.bmj.com/site/about/guidelines.xhtml

The present finding that adults aged 50 years and older with nonrefractive visual impairment accumulated lower lifestyle physical activity than those with normal vision, specifically in women, supports that of previous research and adds to it through the use of objective physical activity measures. For example, in a recent study of 6,634 older English adults those with fair–poor and good eyesight were significantly more likely to be inactive, categorized by self-report, than those who reported excellent eyesight.[14] Further research is needed to understand why older adults with reduced eyesight have lower levels of physical activity. One plausible explanation may be fear of going outside, owing to falling or suffering other accidents. This low level of physical activity is of concern as this population may be at an increased risk of chronic illness, such as higher risk of cancer[25] and also have associated risk factors such as higher smoking rates,[26] independent of physical activity. Moreover, those who are visually impaired often report having a low quality of life.[27]

A high prevalence of sedentary time in those aged 20-49 years with non-refractive visual impairment should be noted. Indeed, the present study has shown higher levels of sedentary time among women with nonrefractive visual impairment compared with those with normal vision. Refractory visual problems can normally be corrected and are likely to be less disabling than nonrefractive visual impairment. Moreover, nonrefractive visual impairment are likely to be comorbid. Therefore, those with nonrefractive visual impairment may be a population with greater barriers to physical activity participation. The lack of association between sedentary time and vision status in older adults is interesting. A plausible explanation is that as adults age, sedentary time is likely to increase regardless of disability status. Therefore, when those with and without visual

For peer review only - http://bmjopen.bmj.com/site/about/guidelines.xhtml

impairment reach older adulthood the difference in time spent sedentary is negligible. A rationale for stronger associations with higher levels of sedentary behavior in women but not men with visual impairment is elusive and further qualitative research to explain this finding is required.

Clear strengths of this study are the large population-based sample of US adolescents and adults and objective measurement of physical activity and sedentary time. However, the data must be interpreted in light of its limitations. Analyses are of a cross-sectional design and thus it is not known whether visual impairment leads to low levels of activity and high levels of sedentary time or vice versa. Indeed, adequate levels of physical activity and lower sedentary time may decrease risk for visual impairment by reducing risk for diabetes and associated complications such as cataracts and diabetic retinopathy.

In conclusion, findings from the present study suggest generally low levels of physical activity and high levels of sedentary time in adolescents. However, activity patterns are similar between adolescents with visual impairment and those with normal vision. Adult women with nonrefractive visual impairment have lower levels of lifestyle physical activity (aged 50+ years) and higher levels of sedentary time (aged 20-49 years) than those with normal vision. Taken together, these findings highlight the need for interventions to promote physical activity and reduce sedentary time in adult populations with visual impairment, specifically adult women.

For peer review only - http://bmjopen.bmj.com/site/about/guidelines.xhtml

REFERENCES

1. Caspersen CJ, Powell KE, Christenson GM. Physical activity, exercise, and physical fitness: definitions and distinctions for health-related research. *Public Health Rep*1985;100:126-31.

2. Warburton DER, Bredin SSD. Health benefits of physical activity: a systematic review of current systematic reviews. *Curr Opin Cardiol* 2017;32:541-556.

3. de Rezende LF, Rodrigues Lopes M, Rey-Lopez JP, et al. Sedentary behavior and health outcomes: an overview of systematic reviews. *PLoS One* 2014;9:e105620.

4. World Health Organization. Global recommendations on physical activity for health. Geneva, Switzerland: World Health Organization 2010.

5. Chief Medical Office (CMO): UK physical activity guidelines.https://www.gov.uk/government/publications/uk-physical-activity-guidelines. Accessed Jun 26 2018., 2011

6. The Department of Health: Australia's Physical Activity and Sedentary Behaviour Guidelines. http://www.health.gov.au/internet/main/publishing.nsf/content/health-pubhlth-strateg-phys-act-guidelines. Accessed Jun 26 2018., 2017

7. Bentley GF, Jago R, Turner KM. Mothers' perceptions of the UK physical activity and sedentary behaviour guidelines for the early years (Start Active, Stay Active): a qualitative study. *BMJ Open* 2015;5:e008383.

8. LeBlanc AG, Berry T, Deshpande S, et al. Knowledge and awareness of Canadian Physical Activity and Sedentary Behaviour Guidelines: a synthesis of existing evidence. Appl *Physiol Nutr Metab* 2015;40:716-24.

9. Tucker JM, Welk GJ, Beyler NK. Physical activity in U.S.: adults compliance with the Physical Activity Guidelines for Americans. *Am J Prev Med* 2011;40:454-61.

For peer review only - http://bmjopen.bmj.com/site/about/guidelines.xhtml

10. Smith L, Ekelund U, Hamer M. The potential yield of non-exercise physical activity energy expenditure in public health. *Sports Med* 2015;45:449-52.

11. Rimmer JH, Riley B, Wang E, et al. Physical activity participation among persons with disabilities: barriers and facilitators. *Am J Prev Med* 2004;26:419-25.

12. Capella-McDonnall M. The need for health promotion for adults who are visually impaired. *J Vis Impair Blind* 2007;101:133-45.

13. Phoenix C, Griffin M, Smith B. Physical activity among older people with sight loss: a qualitative research study to inform policy and practice. *Public health* 2015;129:124-30.

14. Smith L, Timmis MA, Pardhan S, et al. Physical inactivity in relation to self-rated eyesight: cross-sectional analysis from the English Longitudinal Study of Ageing. *BMJ Open Ophthalmol* 2017;1:e000046.

15. Williams G, Aggio D, Stubbs B, et al. Physical activity levels in children with sensory problems: Cross-sectional analyses from the Millennium Cohort Study. *Disabil Health J* 2018;11:58-61.

16. Vitale S, Cotch MF., Sperduto RD. Prevalence of visual impairment in the United States. *Jama* 2006, 295, 2158-63.

17. Sylvia LG, Bernstein EE, Hubbard JL, et al. Practical guide to measuring physical activity. *J Acad Nutr Diet* 2014, 114, 199-208.

18. Willis JR, Jefferys JL, Vitale S, et al. Visual impairment, uncorrected refractive error, and accelerometer-defined physical activity in the United States. *Arch Ophthalmol* 2012;130:329-35.

19. Centers for Disease Control and Prevention: National Health and Nutrition Examination Survey. http://www.cdc.gov/nchs/nhanes.htm. Accessed June 21, 2016.,

For peer review only

For peer review only - http://bmjopen.bmj.com/site/about/guidelines.xhtml

20. Vitale S, Cotch MF, Sperduto RD. Prevalence of visual impairment in the United States. *Jama* 2006;295:2158-63.

21. Melanson EL, Jr., Freedson PS. Validity of the Computer Science and Applications, Inc. (CSA) activity monitor. *Med Sci Sports Exerc* 1995;27:934-40.

22. Crouter SE, DellaValle DM, Haas JD, et al. Validity of ActiGraph 2-regression model, Matthews cut-points, and NHANES cut-points for assessing free-living physical activity. *J Phys Act Health* 2013;10:504-14.

23. Demirturk F, Kaya M. Physical Education Lessons and Activity Status of Visually Impaired and Sighted Adolescents. *Med Sci Monit* 2015;21:3521-7.

24. Marques A, Gaspar de Matos M. Adolescents' physical activity trends over the years: a three-cohort study based on the Health Behaviour in School-aged Children (HBSC) Portuguese survey. *BMJ Open* 2014;4:e006012.

25. Pukkala E, Verkasalo PK, Ojamo M, et al. Visual impairment and cancer: a population-based cohort study in Finland. *Cancer Causes Control* 1999;10:13-20.

26. Zhang X, Kahende J, Fan AZ, et al. Smoking and visual impairment among older adults with age-related eye diseases. *Prev Chronic Dis* 2011;8:A84.

27. Anokye NK, Trueman P, Green C, et al. Physical activity and health related quality of life. *BMC Public Health* 2012;12:624.

Authors Contributions: LY did the analyses. LS and SJ interpreted the results and drafted the manuscript. LS, SEJ, SP, GFLS, LH, CC, DV, AK, BS, JP, and LY critically appraised the manuscript and approved the final version before submission.

For peer review only

For peer review only - http://bmjopen.bmj.com/site/about/guidelines.xhtml

Funding Statement: Sarah Jackson is supported by Cancer Research UK (C1417/A22962). Guillermo Felipe López Sánchez is supported by a postdoctoral fellowship from the Seneca Foundation—Agency for Science and Technology of the Region of Murcia, Spain (20390/PD/17).

Competing Interests Statement: No conflicting relationship exists for any author.

Data availability statement: All data relevant to the study are included in the article or uploaded as supplementary information.

For peer review only

STROBE 2007 (v4) Statement—Checklist of items that should be included in reports of *cross-sectional studies*

Section/Topic	Item #	Recommendation	Reported on page #
**Title and abstract**	1	(*a*) Indicate the study's design with a commonly used term in the title or the abstract	1
		(*b*) Provide in the abstract an informative and balanced summary of what was done and what was found	3-4
**Introduction**			
Background/rationale	2	Explain the scientific background and rationale for the investigation being reported	5-7
Objectives	3	State specific objectives, including any prespecified hypotheses	7
**Methods**			
Study design	4	Present key elements of study design early in the paper	8
Setting	5	Describe the setting, locations, and relevant dates, including periods of recruitment, exposure, follow-up, and data collection	8
Participants	6	(*a*) Give the eligibility criteria, and the sources and methods of selection of participants	8
Variables	7	Clearly define all outcomes, exposures, predictors, potential confounders, and effect modifiers. Give diagnostic criteria, if applicable	9-11
Data sources/ measurement	8*	For each variable of interest, give sources of data and details of methods of assessment (measurement). Describe comparability of assessment methods if there is more than one group	9-11
Bias	9	Describe any efforts to address potential sources of bias	*8*
Study size	10	Explain how the study size was arrived at	8
Quantitative variables	11	Explain how quantitative variables were handled in the analyses. If applicable, describe which groupings were chosen and why	
Statistical methods	12	(*a*) Describe all statistical methods, including those used to control for confounding	12
		(*b*) Describe any methods used to examine subgroups and interactions	12
		(*c*) Explain how missing data were addressed	12
		(*d*) If applicable, describe analytical methods taking account of sampling strategy	12
		(*e*) Describe any sensitivity analyses	12
**Results**			
Participants	13*	(a) Report numbers of individuals at each stage of study—eg numbers potentially eligible, examined for eligibility,	8

For peer review only

		confirmed eligible, included in the study, completing follow-up, and analysed	
		(b) Give reasons for non-participation at each stage	8
		(c) Consider use of a flow diagram	
Descriptive data	14*	(a) Give characteristics of study participants (eg demographic, clinical, social) and information on exposures and potential confounders	8
		(b) Indicate number of participants with missing data for each variable of interest	8
Outcome data	15*	Report numbers of outcome events or summary measures	13
Main results	16	(*a*) Give unadjusted estimates and, if applicable, confounder-adjusted estimates and their precision (eg, 95% confidence interval). Make clear which confounders were adjusted for and why they were included	13-18
		(*b*) Report category boundaries when continuous variables were categorized	13-18
		(*c*) If relevant, consider translating estimates of relative risk into absolute risk for a meaningful time period	
Other analyses	17	Report other analyses done—eg analyses of subgroups and interactions, and sensitivity analyses	13-18
**Discussion**			
Key results	18	Summarise key results with reference to study objectives	18-21
Limitations	19	Discuss limitations of the study, taking into account sources of potential bias or imprecision. Discuss both direction and magnitude of any potential bias	18-21
Interpretation	20	Give a cautious overall interpretation of results considering objectives, limitations, multiplicity of analyses, results from similar studies, and other relevant evidence	18-21
Generalisability	21	Discuss the generalisability (external validity) of the study results	18-21
**Other information**			
Funding	22	Give the source of funding and the role of the funders for the present study and, if applicable, for the original study on which the present article is based	2

*Give information separately for cases and controls in case-control studies and, if applicable, for exposed and unexposed groups in cohort and cross-sectional studies.

Note: An Explanation and Elaboration article discusses each checklist item and gives methodological background and published examples of transparent reporting. The STROBE checklist is best used in conjunction with this article (freely available on the Web sites of PLoS Medicine at http://www.plosmedicine.org/, Annals of Internal Medicine at http://www.annals.org/, and Epidemiology at http://www.epidem.com/). Information on the STROBE Initiative is available at www.strobe-statement.org.

For peer review only

BMJ Open

Visual impairment and objectively measured physical activity and sedentary behavior in US adolescents and adults: a cross-sectional study

Journal:	*BMJ Open*
Manuscript ID	bmjopen-2018-027267.R2
Article Type:	Research
Date Submitted by the Author:	11-Mar-2019
Complete List of Authors:	Smith, Lee; Anglia Ruskin University - Cambridge Campus, Department of Life Sciences<br>Jackson, Sarah ; University College London, Epidemiology and Public Health;<br>Pardhan, Shahina ; Anglia Ruskin University, Vision and Eye Research Unit (VERU), School of Medicine<br>López-Sánchez, Guillermo Felipe; Faculty of Sport Sciences, University of Murcia<br>Hu, Liang; Zhejiang University, Physical Education<br>Chao, Cao; Division of Public Health Sciences, Department of Surgery, Washington University School of Medicine, St Louis, MO, USA; Centre for Public Health Systems Science, Brown School Washington University, St Louis, MO USA.<br>Vancampfort, D.; Department of Rehabilitation Sciences, KU Leuven, University of Leuven, Leuven, Belgium; University Psychiatric Centre, KU Leuven, University of Leuven, Leuven-Kortenberg, Belgium<br>Koyanagi, Ai; Parc Sanitari Sant Joan de Deu, Research and Development Unit<br>Stubbs, B; King's College London<br>Firth, Joseph ; University of Western Sydney, NICM Health Research Institute<br>Yang, Lin; Department of Epidemiology, Medical University of Vienna, Austria
**Primary Subject Heading**:	Epidemiology
Secondary Subject Heading:	Epidemiology
Keywords:	Physical activity, Sedentary behaviour, Accelerometer, Visual impairment, Diabetic retinopathy < DIABETES & ENDOCRINOLOGY, NHANES

SCHOLARONE™
Manuscripts

Visual impairment and objectively measured physical activity and sedentary behavior in US adolescents and adults: a cross-sectional study

Lee Smith[1]*, Sarah E Jackson[2]*, Shahina Pardhan[3], Guillermo Felipe López-Sánchez[4], Liang Hu[5], Chao Cao[6], Davy Vancampfort[7], Ai Koyanagi[8], Brendon Stubbs[9], Joseph Firth[10], Lin Yang[11]

1. The Cambridge Centre for Sport and Exercise Sciences, Anglia Ruskin University, Cambridge

2. Department of Behavioural Science and Health, UCL, London, UK

3. Vision & Eye Research Unit (VERU), School of Medicine, Anglia Ruskin University, Cambridge, UK

4. Faculty of Sport Sciences, University of Murcia, Spain.

5. Department of sport science, Zhejiang University, Hangzhou, China

6.Division of Public Health Sciences, Department of Surgery, Washington University School of Medicine, St Louis, MO, USA; Centre for Public Health Systems Science, Brown School Washington University, St Louis, MO USA.

7. Department of Rehabilitation Sciences, KU Leuven, University of Leuven, Leuven, Belgium; University Psychiatric Centre, KU Leuven, University of Leuven, Leuven-Kortenberg, Belgium;

For peer review only - http://bmjopen.bmj.com/site/about/guidelines.xhtml

8. Research and Development Unit, ParcSanitariSant Joan de Déu, Universitat de Barcelona, FundacióSant Joan de Déu, Dr Antoni Pujadas, 42, SantBoi de Llobregat, Barcelona, 08830, Spain;Instituto de Salud Carlos III, Centro de InvestigaciónBiomédicaen Red de Salud Mental, CIBERSAM, Monforte de Lemos 3-5 Pabellón 11, Madrid, 28029, Spain;

9. Physiotherapy Department, South London and Maudsley NHS Foundation Trust, Denmark Hill, London, UK; Health Service and Population Research Department, Institute of Psychiatry, Psychology and Neuroscience, King's College London, De Crespigny Park, London, UK; Faculty of Health, Social Care and Education, Anglia Ruskin University, Chelmsford, UK.

10. NICM, School of Science and Health, University of Western Sydney, Sydney, Australia; Division of Psychology and Mental Health, Faculty of Biology, Medicine and Health, University of Manchester, Manchester, UK.

11. Department of Epidemiology, Medical University of Vienna, Austria

*These authors share first authorship and are corresponding authors:

Lee Smith: lee.smith@anglia.ac.uk Sarah E Jackson: s.e.jackson@ucl.ac.uk

Word count, excluding title page, abstract, references, figures and tables: 2977.

For peer review only

For peer review only - http://bmjopen.bmj.com/site/about/guidelines.xhtml

ABSTRACT

Objectives: To compare levels of physical activity and sedentary time in a representative sample of US adolescents and adults with and without visual impairment. **Design:** Cross-sectional analyses were carried out using data from the National Health and Nutrition Examination Survey. **Participants:** The study population consisted of 6,001 participants (adolescents n=1,766, adults n=4,235). The present analysis aggregated data from 2003-2004 and 2005-2006. **Measures**: Objective physical activity and sedentary behaviour assessment was conducted over 7 days. Distance visual acuity was measured for each eye in all participants 12 years and older. Participants' vision was categorised as: normal vision, uncorrected refractive error, nonrefractive visual impairment. We estimated the sex-specific linear associations between presenting vision and objectively measured physical activity and sedentary patterns using adjusted generalized linear models in adolescents and adults. **Results and conclusions:** Adolescents with uncorrected refractive error and nonrefractive visual impairment did not accumulate higher levels of sedentary time or lower levels of MVPA compared with those with normal vision. We observed no association between vision status and accelerometer measured MVPA in adults aged 20-49 years. We observed more time spent sedentary among females 20-49 years old with nonrefractive visual impairment compared with those presenting normal vision (mean difference 329.8 mins/week, 95% CI: 12.5 to 647.0). Adults 50 years and older with nonrefractive visual impairment appeared to accumulate less lifestyle physical activity, particularly in women (mean difference -82.8 mins/week, 95% CI: -147.8 to -17.8). Adult women with nonrefractive visual impairment have lower levels of lifestyle physical activity and higher levels of sedentary time than those with normal vision. Taken

For peer review only

For peer review only - http://bmjopen.bmj.com/site/about/guidelines.xhtml

together, these findings highlight the need for interventions to promote physical activity and reduce sedentary time in adult populations with visual impairment, specifically adult women.

Key words: Physical activity, sedentary behaviour, accelerometer, visual impairment, diabetic retinopathy, NHANES.

STRENGTHS AND LIMITATIONS OF THIS STUDY

- Large, population-based sample of US adolescents and adults.
- Objective measures of physical activity and sedentary time.
- Analyses are of a cross-sectional design and thus it is not known whether visual impairment leads to low levels of activity and high levels of sedentary time or vice versa.
- Risk for developing diabetes and associated complications such as cataracts and diabetic retinopathy may be reduced in those with visual impairment by participating in adequate levels of physical activity and lower amounts of sedentary behavior.

For peer review only

For peer review only - http://bmjopen.bmj.com/site/about/guidelines.xhtml

INTRODUCTION

Physical activity may be defined as any bodily movement caused by contraction of skeletal muscle that results in energy expenditure[1] and may include activities such as structured exercise and sport, active travel (walking and cycling), occupational activity, and household chores/gardening. Regular and sustained participation in physical activity is beneficial for almost every facet of health in children, adolescents and adults. For example, it has been associated with lower incidence of cardiovascular disease, cancer and osteoarthritis, and promotes positive mental health in all ages.[2] At the other end of the energy expenditure spectrum, excessive time in sedentary behaviour has been shown to be detrimental to both physical and mental health, and this is independent from physical activity levels.[3]

In light of the positive benefits of regular participation in physical activity and the detrimental effects of sedentary behaviour, guidelines have been developed. The World Health Organisation states that to maintain good health, adults should achieve at least 30 minutes of moderate activity (e.g. brisk walking) on five or more days of the week.[4] Moreover, country-specific guidelines state that sedentary time should be kept to a minimum.[5-8] It is advised that children and adolescents should achieve 60 minutes of moderate activity on each day of the week and keep sedentary time to a minimum.[4] However, despite these recommendations population levels of physical activity are low, particularly in Western countries.[9] Data from the National Health Nutrition and Examinations Survey (NHANES) showed that in 2005-2006 fewer than 10% of US adults met the recommended physical activity guidelines.[9] It is likely that any increase in energy

For peer review only - http://bmjopen.bmj.com/site/about/guidelines.xhtml

expenditure is beneficial to health. It has been hypothesised that increases in physical activity energy expenditure may have health benefits regardless of how that increase is achieved (i.e., achieved via short periods of moderate activity or long periods of light-intensity activity that may yield equivalent levels of energy expenditure).[10] Considering these potential health benefits, all levels of physical activity should be promoted in all populations.

Persons with disabilities have been shown to have low levels of physical activity and high levels of sedentary behaviour and understanding differences versus the general population is important.[11] The disability of reduced eyesight may be a key barrier to an active lifestyle in adults and adolescents. It has been suggested that in people with visual impairment there is a lack of access to recreational and athletic programmes, and help or encouragement in developing suitable and safe physical recreation skills and habits.[12] Moreover, this population experiences activity limitations in walking, and environmental barriers such as transport and lack of accessible exercise equipment can hamper a person's ability to be physically active. [12-13] The authors of the present paper have shown in a sample of 6634 United Kingdom participants (mean age 65.0±9.2 years) those with poor vision were twice as likely to be physically inactive than those with good eyesight. Similar findings were found for the variable 'recognition of friends across street' and 'reading ordinary newspaper'.[14] The present authors have also found similar associations in young people.[15] These findings are of importance owing to a high prevalence of reduced eyesight. For example, in the USA it has been estimated that approximately 14 million individuals aged 12 years or older have visual impairment (defined as distance visual acuity of 20/50 or worse).[16] However, key limitations to previously discussed

For peer review only - http://bmjopen.bmj.com/site/about/guidelines.xhtml

analyses were that crude self-reported measures of vision were used (not allowing one to distinguish between types of eye conditions, refractive and nonrefractive) and physical activity was self-reported. Self-reported physical activity is subject to bias owing to participants not being able to accurately recall physical activity and reporting higher levels of physical activity than the actual truth. Further research is needed using objective measures of vision and objective measures of physical activity behaviour such as accelerometers, which are a more accurate measure of free living physical activity. [17]

One previous study has looked at visual impairment and objective physical activity using the NHANES cohort. This study found that in those older than 20 years, individuals with normal sight took an average of 9964 steps per day and engaged in an average of 23.5 minutes per day of moderate-to-vigorous physical activity (MVPA), as compared with 9742 steps per day and 23.1 minutes per day of MVPA in individuals with uncorrected refractive error ($P > .50$ for both) and 5992 steps per day and 9.3 minutes/d of MVPA in individuals with visual impairment ($P < .01$ for both). In multivariable models, individuals with visual impairment took 26% fewer steps per day ($P < .01$; 95% CI, 18%-34%) and spent 48% less time in MVPA ($P < .01$; 95% CI, 37%-57%) than individuals with normal sight. However, this study did not look at associations between sedentary time, light physical activity and visual impairment, and did not explore levels of activity in adolescence or older adults.[18] Moreover, although this study controlled for the important covariates age, sex, race, obesity, education and systemic disease, it did not control for health behaviours such as smoking which has also previously been shown to be associated with both physical activity[19] and visual impairment.[20]

For peer review only - http://bmjopen.bmj.com/site/about/guidelines.xhtml

146 The present paper therefore aimed to compare levels of physical activity (light, MVPA, lifestyle) and sedentary time in a representative sample of US adolescents, adults, and older adults using objective measures of physical activity and visual impairment controlling for a wide range of demographic and behavioural covariates.

150

151

152

For peer review only

For peer review only - http://bmjopen.bmj.com/site/about/guidelines.xhtml

154 **METHODS**

155 **Study Population**

156 The NHANES was designed to provide cross-sectional estimates of the prevalence of health, nutrition, and potential risk factors among the civilian non-institutionalized U.S. population up to 85 years of age.[21] In brief, NHANES surveys a nationally representative complex, stratified, multistage, probability clustered sample of around 5,000 participants each year in 15 counties across the country. Survey participants are asked to attend a physical examination either in a mobile examination centre (MEC) or in the participants' home. The present analysis aggregated data from 2003-2004 and 2005-2006. During these waves, objective physical activity and sedentary behaviour assessment was implemented in the NHANES participants by fitting them with a hip-worn accelerometer (ActiGraph AM-7164) for 7 days. The NHANES obtained ethical approval from the National Centre for Health Statistics Research Ethics Review Board and participants provided written informed consent.

168 Data on socio-demographic information, measures of adiposity, smoking history, vision examination and objective physical activity were extracted and combined into a single dataset for each data collection wave, 2003-2004 and 2005-2006. Further, participants who were pregnant or had physical functional impairments that limited their ability to crawl, walk, run or play (age 12-19 years old) or limited them from walking for a quarter mile, or walking up ten steps (20 years and above) were excluded.

For peer review only - http://bmjopen.bmj.com/site/about/guidelines.xhtml

Presenting visual acuity

NHANES participants undertook the vision examination at the MEC. The procedure of vision examination has been detailed elsewhere.[22] In brief, distance visual acuity was measured for all participants 12 years and older for each eye. An autorefractor (ARK0760, Nidek Co Ltd, Tokyo, Japan) was used which contains built-in visual acuity charts with 20/20, 20/25, 20/30, 20/40, 20/50, 20/60, 20/80 and 20/200 lines. Presenting visual acuity was defined using the better eyes with participants' usual distance version correction, if any. The 20/50 line was presented first, with at least 4 of the 5 characters to be read correctly to advance to the next line, otherwise the 20/200 line was presented. For eyes with presenting visual acuity of 20/30 or worse, visual acuity were measured after incorporating information from the objective refraction measurement. Participants with better seeing eyes with distance visual acuity of 20/30 or better were categorized as having normal vision. Participants with better-seeing eyes of presenting visual impairment that improved, aided by automated refraction result to 20/40 or better were categorized as having uncorrected refractive error, otherwise nonrefractive visual impairment.[22]

Accelerometer measured activity pattern

NHANES participants were asked during their physical examinations at the MEC to wear an accelerometer (ActiGraph AM-7164, 1-minute epochs) on the right hip for 7 consecutive days to objectively measure free-living physical activity. The ActiGraph AM-7164 is a validated, small lightweight device that provides detailed information about the intensity, frequency and duration of physical activity.[23] The epoch length was set at 1 minute, and the Actigraph recorded data for physical activity in the form of counts per

For peer review only - http://bmjopen.bmj.com/site/about/guidelines.xhtml

minute (cpm). Non-wear time was defined as 60 minutes of consecutive zero counts. A recording of at least 10 hours of data was defined as a valid day, and four or more valid days were required to be included in the analysis. The total minutes of valid data were recorded as the accelerometer wear time. Based on standard cpm cutoff methods[24], four raw activity outcomes were derived: sedentary behaviour (<100 cpm), light intensity physical activity (100-759 cpm), lifestyle activity or ambulatory (760-2109) and at least 10 minutes of moderate-to-vigorous physical activity (>2020 cpm). We further computed wearing time adjusted activity by dividing each raw activity minutes by total wear time and multiplying the resulting fraction by the average wear time of all participants. We summarized the adjusted total weekly minutes of sedentary behaviour, light intensity physical activity, lifestyle physical activity, and MVPA for each participant.

Socio-demographic characteristics

Socio-demographic characteristics including age, sex, race and ethnicity, household income, employment status, and smoking status were extracted. Based on self-reported race and ethnicity, participants were classified into one of the three racial groups: Non-Hispanic white, Non-Hispanic black, and Hispanic and others. Annual household income was grouped into <20,000; 20,000-74999; and ≥75,000 US dollars. Employment status was dichotomized to employed vs. unemployed. We classified participants into three groups: never smokers (did not smoke 100 cigarettes and do not smoke now), former smokers (smoked 100 cigarettes in life and do not smoke now), and current smokers (smoked 100 cigarettes in life and smoke now).

Overweight and obesity criteria

For peer review only - http://bmjopen.bmj.com/site/about/guidelines.xhtml

219 Weight and height were measured during the physical examination in MEC or in the
220 participant's home for those whose travel was limited. The measurements followed
221 standard procedures and were carried out by trained technicians with standardized
222 equipment. BMI was calculated as weight in kg / (height in meters)^2. The standard
223 definition for overweight and obesity classification was used to divide the BMI values into
224 three categories: underweight or normal weight (<25.0), overweight (25.0 – 29.9), and
225 obese (≥30.0).

226 **Chronic illness**

227 We included four chronic conditions: cardiovascular diseases, diabetes, cancer and
228 arthritis. Participants were considered as having chronic illness if they self-reported being
229 told by a physician that they have the following conditions: congestive heart failure,
230 coronary heart disease, heart attack, a stroke (cardiovascular diseases), diabetes,
231 cancer, or arthritis.

232 **Patient and Public Involvement**

233 Patients and the public were not involved in the design of the present study.

234

For peer review only

235 **Statistical Analysis**

236 Survey analysis procedures were used to account for the sample weights, stratification, and clustering of the complex sampling design to ensure nationally representative estimates. Descriptive statistics for participant characteristics were calculated by presenting vision. Sample size and weighted proportions were summarized.

240 Sex-specific linear associations between presenting vision (normal vision, uncorrected refractive error, nonrefractive visual impairment) and objectively measured physical activity and sedentary patterns were estimated using generalized linear models in children and adolescents aged between 12-19 years old, adult 20-49 years and older adults aged 50+ years, respectively. Generalized linear models were adjusted for age, race, BMI and household income among those between 12-19 years old, and additionally adjusted for employment status, smoking, and chronic illness among adults (20-49 years, and 50 years and older). Due to the small amount of light intensity physical activity and lifestyle physical activity in the younger population, we only included sedentary time and MVPA in models for population age 12-19 years old.

250 Finally, marginal means were estimated in multivariate adjusted generalized linear models for each modelled outcomes of accelerometer measured activity pattern. All statistical significance was set at $p<0.05$. All statistical analyses were performed using Stata version 14.0 (STATA Corp., College Station, Texas, USA).

For peer review only

For peer review only - http://bmjopen.bmj.com/site/about/guidelines.xhtml

RESULTS

The study population consisted of 6,001 participants who had data on presenting refractive error and accelerometer measured activity pattern. The majority of the participants were 20 years and older (n=4235). Overall, 60.8% (weighted proportion) of participants had presenting normal vision, 33.8% and 5.4% had uncorrected refractive error or non-refractive visual impairment. Participants who were older, female, unemployed, with education high school or lower and chronic conditions were more likely to have nonrefractive visual impairment compared with younger, male, employed, well-educated participants and those with no chronic illness conditions. (Table 1).

Tables 2 - 4 summarize both the non-adjusted and adjusted associations between presenting vision status and accelerometer measured activity pattern in younger (12-19 years old) participants and adults aged 20-49 years and ≥50 years, respectively. Children and adolescents aged between 12-19 years old with uncorrected refractive error and nonrefractive visual impairment did not accumulate higher levels of sedentary time or lower levels of MVPA compared with those with normal vision. The null association was seen in both male and female. After adjusting for age, BMI, race and household income, the estimated marginal mean of sedentary time in those with nonrefractive visual impairment were 3982.7 minutes per week (equivalent 9.5 hours a day) and 3722.9 minutes per week (equivalent 8.8 hours a day) in male and female, respectively. The estimated marginal mean of MVPA in those with nonrefractive visual impairment were 156 minutes per week in male and 55 minutes per week in female.

For peer review only - http://bmjopen.bmj.com/site/about/guidelines.xhtml

Table 1. Socio-demographic characteristics and objectively measured physical activity level of US population aged 12 and older from the NHANES (2003-2006) by visual impairment

	Normal vision		Uncorrected refractive error		Visual impairment		
	N	Weighted %	N	Weighted %	N	Weighted %	p
Overall	3350	60.8%	2225	33.8%	426	5.4%	
Weighted N	70084202		38937983		6184612		
Age group, y							0.001
12-19	999	11.2%	689	12.4%	78	6.8%	
20 - 49	1394	57.0%	620	42.1%	58	21.9%	
≥50	937	31.8%	916	44.5%	290	71.3%	
Sex							0.004
Men	1837	53.5%	1114	47.0%	223	49.9%	
Women	1513	46.5%	1111	53.0%	203	50.1%	
Race/ethnicity							0.257
Non-Hispanic white	1555	74.5%	950	71.3%	229	77.0%	
Non-Hispanic black	802	9.6%	543	10.7%	86	9.5%	
Hispanic	993	15.9%	732	18.0%	111	13.5%	
Other	133	4.8%	91	5.7%	8	3.8%	
Household income							<.001
< 20,000	964	17.5%	806	24.7%	209	38.6%	
20000-74999	1740	52.2%	1162	53.5%	218	56.8%	
≥75,000	976	36.5%	511	31.6%	56	18.0%	
Weight status[a]							0.649
Normal	1416	37.1%	989	39.4%	171	37.5%	
Overweight	1009	32.4%	655	32.3%	141	32.0%	
Obesity	911	30.0%	571	27.9%	112	30.1%	
Employment status[b]							
Unemployed	664	23.0%	724	35.9%	242	59.0%	
Employed	1604	77.0%	780	64.1%	98	41.0%	
Smoking status[b]							0.079
Never smoker	1243	46.0%	780	45.0%	167	44.6%	

For peer review only - http://bmjopen.bmj.com/site/about/guidelines.xhtml

Former smoker	611	22.7%	450	24.2%	130	33.3%	
Current smoker	496	20.0%	306	18.4%	51	15.3%	
Chronic condition[c]							<.001
Yes	649	23.1%	616	30.3%	180	44.4%	

[a]Body mass index was calculated as weight in kilograms divided by height in meters squared. Weight status were defined using BMI-to-age percentile in 12-19 years old, and WHO standard cut-off for adults 20 years and older

[b]Data are only available on adults age 20 years and older

[c]If participants have one of the following conditions: cardiovascular disease, diabetes, arthritis, and cancer

For peer review only

Table 2. Accelerometer measured activity pattern (minutes per week) by vision status in the NHANES (2003-2006) children and adolescents aged 12-19 years

	Sedentary time									
	Male					Female				
	Un-adjusted β (95%CI)		MV-adjusted β (95%CI)[a]		Marginal mean	Un-adjusted β (95%CI)		MV-adjusted β (95%CI)[a]		Marginal mean
Normal vision	Ref		Ref		3354.3	Ref		Ref		3541.5
Uncorrected refractive error	-50.9	(-326.4 to224.5)	-101.7	(-362.5 to159.2)	3252.6	-63.3	(-257.0 to130.4)	-41.4	(-226.85 to 144.1)	3500.1
Visual impairment	697.5	(-326.4 to1721.3)	628.5	(-296.6 to1553.5)	3982.7	141.5	(-424.5 to707.5)	181.4	(-347.99 to 710.8)	3722.9
P trend	0.578		0.815			0.844		0.960		

	Moderate to vigorous physical activity									
	Male					Female				
	Un-adjusted β (95%CI)		MV-adjusted β (95%CI)[a]		Marginal mean	Un-adjusted β (95%CI)		MV-adjusted β (95%CI)[a]		Marginal mean
Normal vision	Ref		Ref		104.8	Ref		Ref		47.7
Uncorrected refractive error	-1.4	(-24.2 to21.4)	-9.1	(-30.0 to11.8)	95.7	-4.3	(-19.6 to11.0)	-5.7	(-21.4 to10.1)	42.0
Visual impairment	63.9	(-1.2 to129.1)	51.3	(-16.4 to119.0)	156.1	10.2	(-36.9 to57.2)	7.7	(-36.7 to52.1)	55.4
P trend	0.432		0.870			0.888		0.759		

[a] Multivariate adjusted models included age (continuous), BMI, race and household income

For peer review only - http://bmjopen.bmj.com/site/about/guidelines.xhtml

Table 3. Accelerometer measured activity pattern (minutes per week) by vision status in the NHANES (2003-2006) adults aged 20 -49 years

	Male					Female				
**Sedentary time**	Un-adjusted β (95%CI)		MV-adjusted β (95%CI)[a]		Marginal mean	Un-adjusted β (95%CI)		MV-adjusted β (95%CI)[a]		Marginal mean
Normal vision	Ref		Ref		3367.7	Ref		Ref		3326.2
Uncorrected refractive error	-55.8	(-213.0 to 101.4)	-13.5	(-180.8 to 153.9)	3354.2	-5.0	(-184.5 to 174.5)	9.9	(-193.6 to 213.4)	3336.1
Visual impairment	52.7	(-496.7 to 602.2)	273.9	(-323.3 to 871.0)	3641.5	257.9	(-90.8 to 606.5)	329.8	(12.5 to 647.0)	3655.9
P trend		0.646		0.659			0.666		0.500	
**Light intensity physical activity**	Un-adjusted β (95%CI)		MV-adjusted β (95%CI)[a]		Marginal mean	Un-adjusted β (95%CI)		MV-adjusted β (95%CI)[a]		Marginal mean
Normal vision	Ref		Ref		1871.5	Ref		Ref		1900.1
Uncorrected refractive error	41.3	(-71.8 to 154.3)	25.1	(-86.5 to 136.8)	1896.6	23.7	(-84.1 to 131.5)	6.5	(-101.6 to 114.7)	1906.7
Visual impairment	292.8	(-37.5 to 623.1)	7.3	(-77.9 to 505.1)	2085.1	-5.7	(-256.4 to245.0)	-15.5	(-264.5 to 233.4)	1884.6
P trend		0.047		0.198			0.717		0.953	
**Lifestyle physical activity**	Un-adjusted β (95%CI)		MV-adjusted β (95%CI)[a]		Marginal mean	Un-adjusted β (95%CI)		MV-adjusted β (95%CI)[a]		Marginal mean
Normal vision	Ref		Ref		815.9	Ref		Ref		654.5
Uncorrected refractive error	9.9	(-55.74 to 75.5)	-2.4	(-70.1 to 65.3)	813.5	-13.3	(-59.6 to 33.1)	-15.0	(-60.6 to 30.6)	639.5
Visual impairment	115.9	(-112.8 to 344.5)	45.9	(-157.2 to 249.1)	861.8	-68.6	(-187.6 to- 50.3)	-81.3	(-181.7 to 19.0)	573.2
P trend		0.330		0.842			0.337		0.249	
**Moderate to vigorous physical activity**	Un-adjusted β (95%CI)		MV-adjusted β (95%CI)[a]		Marginal mean	Un-adjusted β (95%CI)		MV-adjusted β (95%CI)[a]		Marginal mean

For peer review only - http://bmjopen.bmj.com/site/about/guidelines.xhtml

Normal vision	Ref		Ref		62.6	Ref		Ref		49.8
Uncorrected refractive error	11.4	(-4.2 to 27.0)	10.2	(-6.8 to 27.2)	72.9	2.1	(-8.4 to 12.6)	-2.9	(-11.5 to 5.7)	46.9
Visual impairment	2.7	(-28.3 to 33.7)	3.9	(-25.9 to 33.7)	66.5	1.7	(-29.3 to 32.7)	-2.7	(-25.5 to 20.1)	47.1
P trend		0.226		0.287			0.624		0.457	

Multivariate adjusted models included age (continuous), BMI, race , household income, employment status, smoking status, and chronic illness

For peer review only

Table 4. Accelerometer measured activity pattern (minutes per week) by vision status in the NHANES (2003-2006) adults aged 50 years and older

Sedentary time

	Male					Female				
	Un-adjusted β (95%CI)		MV-adjusted β (95%CI)[a]		Marginal mean	Un-adjusted β (95%CI)		MV-adjusted β (95%CI)[a]		Marginal mean
Normal vision	Ref		Ref		3756.2	Ref		Ref		3502.5
Uncorrected refractive error	-42.0	(-196.9 to 112.9)	-100.7	(-271.5 to 70.0)	3655.5	211.0	(47.9 to 374.2)	123.8	(-52.4 to 300.0)	3626.3
Visual impairment	290.9	(-26.9 to 608.7)	230.8	(-183.3 to 513.0)	3921.0	318.3	(44.4 to 592.2)	179.6	(-104.9 to 464.1)	3682.1
P trend	0.244		0.816			0.003		0.098		

Light intensity physical activity

	Male					Female				
	Un-adjusted β (95%CI)		MV-adjusted β (95%CI)[a]		Marginal mean	Un-adjusted β (95%CI)		MV-adjusted β (95%CI)[a]		Marginal mean
Normal vision	Ref		Ref		1815.6	Ref		Ref		1914.4
Uncorrected refractive error	-202.7	(-330.4 to -75.0)	-134.9	(-257.6 to -12.2)	1680.7	-115.9	(-187.5 to -44.3)	-34.9	(-103.6 to 24.8)	1875.0
Visual impairment	-196.0	(-311.91 to -80.1)	-57.2	(-182.2 to 67.81)	1758.4	-194.8	(-335.3 to -54.2)	-56.6	(-205.9 to 92.7)	1857.8
P trend	< .001		0.063			0.001		0.234		

Lifestyle physical activity

	Male					Female				
	Un-adjusted β (95%CI)		MV-adjusted β (95%CI)[a]		Marginal mean	Un-adjusted β (95%CI)		MV-adjusted β (95%CI)[a]		Marginal mean
Normal vision	Ref		Ref		632.2	Ref		Ref		507.2
Uncorrected refractive error	-126.6	(-190.4 to -62.8)	-54.4	(-106.1 to -2.7)	577.8	-103.5	(-143.2 to -63.8)	-41.0	(-79.8 to -2.3)	466.1
Visual impairment	-224.1	(-298.9 to -149.2)	-56.5	(-140.0 to 27.0)	575.6	-221.0	(-269.2 to -172.8)	-82.8	(-147.8 to -17.8)	424.4
P trend	< .001		0.051			< .001		0.009		

Moderate to vigorous physical activity

	Male					Female				
	Un-adjusted β (95%CI)		MV-adjusted β (95%CI)[a]		Marginal mean	Un-adjusted β (95%CI)		MV-adjusted β (95%CI)[a]		Marginal mean

For peer review only - http://bmjopen.bmj.com/site/about/guidelines.xhtml

Normal vision	Ref		Ref		48.1	Ref		Ref		30.3
Uncorrected refractive error	-9.2	(-23.3 to 4.9)	-5.0	(-21.5 to 9.4)	42.1	-4.4	(-17.1 to 8.45)	3.4	(-8.6 to 15.4)	33.7
Visual impairment	-16.7	(-43.7 to 10.3)	-5.0	(-36.6 to 26.7)	43.2	0.2	(-21.8 to 22.3)	13.9	(-10.6 to 38.5)	44.2
P trend		0.127		0.579			0.762		0.299	

[a] Multivariate adjusted models included age (continuous), BMI, race , household income, employment status, smoking status, and chronic illness

For peer review only

For peer review only - http://bmjopen.bmj.com/site/about/guidelines.xhtml

Similarly, no association was observed between presenting vision status and accelerometer measured MVPA in adults aged 20-49 years or those aged ≥50 years, although the multivariate marginal mean of MVPA among those aged ≥50 years with nonrefractive visual impairment (48.1 minutes per week in male, 30.3 minutes per week in female) appeared lower than that in the young adult population (62.6 minutes per week in male, 49.8 minutes per week in female). With respect to sedentary behaviour, higher level of sedentary time was observed among women 20-49 years with nonrefractive visual impairment compared with those with presenting normal vision (mean difference mins/week 329.8, 95% CI: 12.5 to 647.0). No association was observed between presenting vision and light intently physical activity in either gender. However, adults 50 years and older with nonrefractive visual impairment appeared to accumulate lower lifestyle physical activity, particularly in women (mean difference mins/week -82.8, 95% CI: -147.8 to -17.8).

DISCUSSION

In the present study of a large population based sample of the US we found that those 12-19 years old with uncorrected refractive error and nonrefractive visual impairment had similar levels of activity and sedentary time to those presenting with normal vision. Among adults aged 20-49 years we found higher levels of sedentary time among women with nonrefractive visual impairment compared with those with normal vision. Moreover, adults aged 50 years and older with nonrefractive visual impairment appeared to accumulate lower lifestyle physical activity, particularly in women.

For peer review only - http://bmjopen.bmj.com/site/about/guidelines.xhtml

For peer review only

The finding that visually impaired adolescents (uncorrected refractive error and nonrefractive visual impairment) exhibit little difference in their level of activity and sedentary behavior compared with adolescents with “normal” vision is interesting. To our knowledge, just one previous study has compared levels of activity in visually impaired adolescents (a combination of uncorrected refractive error and nonrefractive visual impairment) with those with normal vision, and it reported similar findings. In a sample of 53 adolescents completing the International Physical Activity Questionnaire – Short Form, physical activity levels of visually impaired adolescents and sighted adolescents were similar ($p>0.05$).[25] Findings from the present study add to this work through utilizing a large, population-based sample and objective measures of physical activity. Moreover, the present study is the first to compare differences in sedentary time between adolescents with impaired vision and normal sight. One plausible reason for these findings is that adolescents with normal vision have very low levels of physical activity;[26] therefore minimizing any difference in a reduction of physical activity owing to a disability (e.g. reduced eyesight). Another reason for the lack of association could be owing to physical education. Indeed, all adolescents regardless of visual impairment are required to partake in physical education and thus acquire similar levels of physical activity during the school day. The present study found that the estimated marginal mean of MVPA in those with nonrefractive visual impairment was low, 156 minutes per week in males and 55 minutes per week in females, and sedentary time high. Interventions are needed to promote physical activity in adolescents with visual impairments.

The present finding that adults aged 50 years and older with nonrefractive visual impairment accumulated lower lifestyle physical activity than those with normal vision,

For peer review only - http://bmjopen.bmj.com/site/about/guidelines.xhtml

specifically in women, supports that of previous research and adds to it through the use of objective physical activity measures. For example, in a recent study of 6,634 older English adults those with fair–poor and good eyesight were significantly more likely to be inactive, categorized by self-report, than those who reported excellent eyesight.[14] Further research is needed to understand why older adults with reduced eyesight have lower levels of physical activity. One plausible explanation may be fear of going outside, owing to falling or suffering other accidents. This low level of physical activity is of concern as this population may be at an increased risk of chronic illness, such as higher risk of cancer[25] and also have associated risk factors such as higher smoking rates,[27] independent of physical activity. Moreover, those who are visually impaired often report having a low quality of life.[28]

A high prevalence of sedentary time in those aged 20-49 years with non-refractive visual impairment should be noted. Indeed, the present study has shown higher levels of sedentary time among women with nonrefractive visual impairment compared with those with normal vision. Refractory visual problems can normally be corrected and are likely to be less disabling than nonrefractive visual impairment. Moreover, nonrefractive visual impairment are likely to be comorbid. Therefore, those with nonrefractive visual impairment may be a population with greater barriers to physical activity participation. The lack of association between sedentary time and vision status in older adults is interesting. A plausible explanation is that as adults age, sedentary time is likely to increase regardless of disability status. Therefore, when those with and without visual impairment reach older adulthood the difference in time spent sedentary is negligible. A rationale for stronger associations with higher levels of sedentary behavior in women but not men with

For peer review only - http://bmjopen.bmj.com/site/about/guidelines.xhtml

visual impairment is elusive and further qualitative research to explain this finding is required.

Clear strengths of this study are the large population-based sample of US adolescents and adults and objective measurement of physical activity and sedentary time. Moreover, our statistical models controlled for a wide range of demographic and behavioral covariates. However, the data must be interpreted in light of its limitations. Analyses are of a cross-sectional design and thus it is not known whether visual impairment leads to low levels of activity and high levels of sedentary time or vice versa. Indeed, adequate levels of physical activity and lower sedentary time may decrease risk for visual impairment by reducing risk for diabetes and associated complications such as cataracts and diabetic retinopathy. The limited collected information on visual impairment (e.g. lack of information on eye disease such as cataracts or macular degeneration) and the length of time one has been visually impaired may have introduced bias into the analyses. Future research should consider collecting data on specific eye conditions and length of time visual impairment has been present.

In conclusion, findings from the present study suggest generally low levels of physical activity and high levels of sedentary time in adolescents. However, activity patterns are similar between adolescents with visual impairment and those with normal vision. Adult women with nonrefractive visual impairment have lower levels of lifestyle physical activity (aged 50+ years) and higher levels of sedentary time (aged 20-49 years) than those with normal vision. Taken together, these findings highlight the need for interventions to

For peer review only

For peer review only - http://bmjopen.bmj.com/site/about/guidelines.xhtml

promote physical activity and reduce sedentary time in adult populations with visual impairment, specifically adult women.

REFERENCES

1. Caspersen CJ, Powell KE, Christenson GM. Physical activity, exercise, and physical fitness: definitions and distinctions for health-related research. *Public Health Rep*1985;100:126-31.

2. Warburton DER, Bredin SSD. Health benefits of physical activity: a systematic review of current systematic reviews. *Curr Opin Cardiol* 2017;32:541-556.

3. de Rezende LF, Rodrigues Lopes M, Rey-Lopez JP, et al. Sedentary behavior and health outcomes: an overview of systematic reviews. *PLoS One* 2014;9:e105620.

4. World Health Organization. Global recommendations on physical activity for health. Geneva, Switzerland: World Health Organization 2010.

5. Chief Medical Office (CMO): UK physical activity guidelines.https://www.gov.uk/government/publications/uk-physical-activity-guidelines. Accessed Jun 26 2018., 2011

6. The Department of Health: Australia's Physical Activity and Sedentary Behaviour Guidelines. http://www.health.gov.au/internet/main/publishing.nsf/content/health-pubhlth-strateg-phys-act-guidelines. Accessed Jun 26 2018., 2017

7. Bentley GF, Jago R, Turner KM. Mothers' perceptions of the UK physical activity and sedentary behaviour guidelines for the early years (Start Active, Stay Active): a qualitative study. *BMJ Open* 2015;5:e008383.

For peer review only

For peer review only - http://bmjopen.bmj.com/site/about/guidelines.xhtml

8. LeBlanc AG, Berry T, Deshpande S, et al. Knowledge and awareness of Canadian Physical Activity and Sedentary Behaviour Guidelines: a synthesis of existing evidence. Appl *Physiol Nutr Metab* 2015;40:716-24.

9. Tucker JM, Welk GJ, Beyler NK. Physical activity in U.S.: adults compliance with the Physical Activity Guidelines for Americans. *Am J Prev Med* 2011;40:454-61.

10. Smith L, Ekelund U, Hamer M. The potential yield of non-exercise physical activity energy expenditure in public health. *Sports Med* 2015;45:449-52.

11. Rimmer JH, Riley B, Wang E, et al. Physical activity participation among persons with disabilities: barriers and facilitators. *Am J Prev Med* 2004;26:419-25.

12. Capella-McDonnall M. The need for health promotion for adults who are visually impaired. *J Vis Impair Blind* 2007;101:133-45.

13. Phoenix C, Griffin M, Smith B. Physical activity among older people with sight loss: a qualitative research study to inform policy and practice. *Public health* 2015;129:124-30.

14. Smith L, Timmis MA, Pardhan S, et al. Physical inactivity in relation to self-rated eyesight: cross-sectional analysis from the English Longitudinal Study of Ageing. *BMJ Open Ophthalmol* 2017;1:e000046.

15. Williams G, Aggio D, Stubbs B, et al. Physical activity levels in children with sensory problems: Cross-sectional analyses from the Millennium Cohort Study. *Disabil Health J* 2018;11:58-61.

16. Vitale S, Cotch MF., Sperduto RD. Prevalence of visual impairment in the United States. *Jama* 2006, 295, 2158-63.

17. Sylvia LG, Bernstein EE, Hubbard JL, et al. Practical guide to measuring physical activity. *J Acad Nutr Diet* 2014, 114, 199-208.

For peer review only - http://bmjopen.bmj.com/site/about/guidelines.xhtml

18. Willis JR, Jefferys JL, Vitale S, et al. Visual impairment, uncorrected refractive error, and accelerometer-defined physical activity in the United States. *Arch Ophthalmol* 2012;130:329-35.

19. Heydari G, Hosseini M, Yousefifard M, et al. Smoking and physical activity in healthy adults: A cross-sectional study in Tehran. *Tanaffos* 2015;14(4),238-45.

20. Zhang X, Kahende J, Fan AZ, et al. Smoking and visual impairment among older adults with age-related eye diseases. *Prev Chronic Dis* 2011;8(4),A84.

21. Centers for Disesae Control and Prevention: National Health and Nutrition Examination Survey. http://www.cdc.gov/nchs/nhanes.htm. Accessed June 21, 2016.,

22. Vitale S, Cotch MF, Sperduto RD. Prevalence of visual impairment in the United States. *Jama* 2006;295:2158-63.

23. Melanson EL, Jr., Freedson PS. Validity of the Computer Science and Applications, Inc. (CSA) activity monitor. *Med Sci Sports Exerc* 1995;27:934-40.

24. Crouter SE, DellaValle DM, Haas JD, et al. Validity of ActiGraph 2-regression model, Matthews cut-points, and NHANES cut-points for assessing free-living physical activity. *J Phys Act Health* 2013;10:504-14.

25. Demirturk F, Kaya M. Physical Education Lessons and Activity Status of Visually Impaired and Sighted Adolescents. *Med Sci Monit* 2015;21:3521-7.

26. Marques A, Gaspar de Matos M. Adolescents' physical activity trends over the years: a three-cohort study based on the Health Behaviour in School-aged Children (HBSC) Portuguese survey. *BMJ Open* 2014;4:e006012.

27. Zhang X, Kahende J, Fan AZ, et al. Smoking and visual impairment among older adults with age-related eye diseases. *Prev Chronic Dis* 2011;8:A84.

For peer review only - http://bmjopen.bmj.com/site/about/guidelines.xhtml

28. Anokye NK, Trueman P, Green C, et al. Physical activity and health related quality of life. *BMC Public Health* 2012;12:624.

Authors Contributions: LY did the analyses. LS and SJ interpreted the results and drafted the manuscript. LS, SEJ, SP, GFLS, LH, CC, DV, AK, BS, JF, and LY critically appraised the manuscript and approved the final version before submission.

Funding Statement: Sarah Jackson is supported by Cancer Research UK (C1417/A22962). Guillermo Felipe López Sánchez is supported by a postdoctoral fellowship from the Seneca Foundation—Agency for Science and Technology of the Region of Murcia, Spain (20390/PD/17).

Competing Interests Statement: No conflicting relationship exists for any author.

Data availability statement: All data relevant to the study are included in the article or uploaded as supplementary information.

For peer review only - http://bmjopen.bmj.com/site/about/guidelines.xhtml

STROBE 2007 (v4) Statement—Checklist of items that should be included in reports of *cross-sectional studies*

Section/Topic	Item #	Recommendation	Reported on page #
**Title and abstract**	1	(*a*) Indicate the study's design with a commonly used term in the title or the abstract	1
		(*b*) Provide in the abstract an informative and balanced summary of what was done and what was found	3-4
**Introduction**			
Background/rationale	2	Explain the scientific background and rationale for the investigation being reported	5-7
Objectives	3	State specific objectives, including any prespecified hypotheses	7
**Methods**			
Study design	4	Present key elements of study design early in the paper	8
Setting	5	Describe the setting, locations, and relevant dates, including periods of recruitment, exposure, follow-up, and data collection	8
Participants	6	(*a*) Give the eligibility criteria, and the sources and methods of selection of participants	8
Variables	7	Clearly define all outcomes, exposures, predictors, potential confounders, and effect modifiers. Give diagnostic criteria, if applicable	9-11
Data sources/ measurement	8*	For each variable of interest, give sources of data and details of methods of assessment (measurement). Describe comparability of assessment methods if there is more than one group	9-11
Bias	9	Describe any efforts to address potential sources of bias	*8*
Study size	10	Explain how the study size was arrived at	8
Quantitative variables	11	Explain how quantitative variables were handled in the analyses. If applicable, describe which groupings were chosen and why	
Statistical methods	12	(*a*) Describe all statistical methods, including those used to control for confounding	12
		(*b*) Describe any methods used to examine subgroups and interactions	12
		(*c*) Explain how missing data were addressed	12
		(*d*) If applicable, describe analytical methods taking account of sampling strategy	12
		(*e*) Describe any sensitivity analyses	12
**Results**			
Participants	13*	(a) Report numbers of individuals at each stage of study—eg numbers potentially eligible, examined for eligibility,	8

For peer review only

		confirmed eligible, included in the study, completing follow-up, and analysed	
		(b) Give reasons for non-participation at each stage	8
		(c) Consider use of a flow diagram	
Descriptive data	14*	(a) Give characteristics of study participants (eg demographic, clinical, social) and information on exposures and potential confounders	8
		(b) Indicate number of participants with missing data for each variable of interest	8
Outcome data	15*	Report numbers of outcome events or summary measures	13
Main results	16	(*a*) Give unadjusted estimates and, if applicable, confounder-adjusted estimates and their precision (eg, 95% confidence interval). Make clear which confounders were adjusted for and why they were included	13-18
		(*b*) Report category boundaries when continuous variables were categorized	13-18
		(*c*) If relevant, consider translating estimates of relative risk into absolute risk for a meaningful time period	
Other analyses	17	Report other analyses done—eg analyses of subgroups and interactions, and sensitivity analyses	13-18
**Discussion**			
Key results	18	Summarise key results with reference to study objectives	18-21
Limitations	19	Discuss limitations of the study, taking into account sources of potential bias or imprecision. Discuss both direction and magnitude of any potential bias	18-21
Interpretation	20	Give a cautious overall interpretation of results considering objectives, limitations, multiplicity of analyses, results from similar studies, and other relevant evidence	18-21
Generalisability	21	Discuss the generalisability (external validity) of the study results	18-21
**Other information**			
Funding	22	Give the source of funding and the role of the funders for the present study and, if applicable, for the original study on which the present article is based	2

*Give information separately for cases and controls in case-control studies and, if applicable, for exposed and unexposed groups in cohort and cross-sectional studies.

Note: An Explanation and Elaboration article discusses each checklist item and gives methodological background and published examples of transparent reporting. The STROBE checklist is best used in conjunction with this article (freely available on the Web sites of PLoS Medicine at http://www.plosmedicine.org/, Annals of Internal Medicine at http://www.annals.org/, and Epidemiology at http://www.epidem.com/). Information on the STROBE Initiative is available at www.strobe-statement.org.

For peer review only
